# Development of a cross-protective subunit cocktail vaccine against diverse serotypes of *Glaesserella parasuis* infection in pigs

**DOI:** 10.1186/s13567-026-01846-3

**Published:** 2026-09-27

**Authors:** Fengyang Li, Yan Gong, Ziheng Li, Zhen Wang, Zengshuai Wu, Di Zhang, Peng Zhang, Zhichao Lu, Hong Chu, Kaixin Zhang, Diansong Li, Na Li, Liancheng Lei

**Affiliations:** 1 State Key Laboratory for Diagnosis and Treatment of Severe Zoonotic Infectious Diseases, Key Laboratory for Zoonosis Research of the Ministry of Education, Institute of Zoonosis, and College of Veterinary Medicine, Jilin University, Xi’an Road 5333, Changchun, China; 2 College of Animal Science, Yangtze University, Jingzhou, China; 3 Changchun Institute of Biological Products Co.,Ltd., Changchun, China

**Keywords:** Subunit vaccine, *Glaesserella parasuis*, Glässer’s disease, PtsG, GAPDH, VacJ

## Abstract

Glässer’s disease, caused by *Glaesserella parasuis* (GPS), causes substantial economic losses in the swine industry worldwide. This situation is largely attributable to the poor cross-protective efficacy of currently available vaccines. In this study, a multi-antigen cocktail subunit vaccine against GPS infection was developed. In silico antigenicity analysis, the immunoprotective efficacy, and antibody titers of 14 GPS antigens were assessed in a murine model. Finally, VacJ, PtsG, and GAPDH were identified as the component of the cocktail vaccine, with Gel-01 as the optimal adjuvant. Immunization of the cocktail vaccine induced robust humoral and cellular immune responses. The vaccine conferred effective cross-protection against lethal-dose GPS4 and GPS5 infections in mice. As supported by a pilot study, it also demonstrated favorable immunoprotective efficacy against GPS infection in piglets. Furthermore, immune sera from vaccinated mice significantly inhibited the growth of GPS4 and GPS5. Overall, these results suggested that the subunit cocktail vaccine established in this study exhibits great potential for preventing GPS infection under experimental conditions, and further large‑scale pig trials are still required to validate its practical applicability in field production.

**Supplementary Information:**

The online version contains supplementary material available at https://doi.org/10.1186/s13567-026-01846-3.

## Introduction

Porcine Glässer’s disease is a swine-specific bacterial infection that is caused by *Glaesserella parasuis* (GPS) and characterized by meningitis, polyserositis, arthritis, and pneumonia [1]. GPS primarily affects young pigs aged from 4 weeks to 4 months, particularly those at 4–8 weeks of age [2, 3]. Epidemiological investigations show the disease prevalence generally ranges from 10% to 15%, and in severe outbreaks, the mortality rate can escalate to 50% [4]. Thus, GPS poses a severe threat to the health of piglets and fattening pigs. To date, 15 serotypes of GPS have been identified, among which serotypes 4 and 5 are the predominant circulating strains in China, followed by serotypes 12, 13, and 14 [4]. Notably, GPS frequently engages in coinfections with other viral and bacterial pathogens. Common respiratory coinfection pathogens include porcine circovirus type 2 (PCV2), porcine reproductive and respiratory syndrome virus (PRRSV), *Streptococcus suis* (SS), and *Actinobacillus pleuropneumoniae* (APP) [5–7]. Such mixed infections increase morbidity and mortality and result in substantial economic losses that hinder sustainable development of swine industry.

Currently, vaccination remains the primary strategy for preventing swine Glässer’s disease, with available vaccines predominantly categorized into inactivated vaccines and subunit vaccines. Among these, multiple inactivated vaccines have been commercialized globally, which demonstrate favorable protective efficacy against the same serotype(s) [1, 8]. Notably, some commercial inactivated vaccines can provide partial cross-protection against several prevalent heterologous serotypes under field conditions [1]. Nevertheless, their protective spectrum is still restricted by serotype specificity. Cross-protection against non-vaccine serotypes is highly variable and remains limited under large-scale swine production [1]. Additionally, commercial inactivated vaccines have inherent limitations, such as short duration of protection and limited immunogenicity [8]. Therefore, it is urgent to develop a vaccine with robust cross-protection against multiple serotypes of GPS infections.

Subunit vaccines, composed of immunoprotective components derived from microbial secretory proteins or outer membrane proteins, offer prominent advantages of high safety and minimal side effects [8, 9]. Of note, only two bivalent subunit vaccines (SS-GPS and PCV2-GPS, both of which use the phenylalanine ammonia-lyase family protein PalA as the primary antigen) were approved for market use [1]. Moreover, a series of multi-epitope vaccines against GPS have been reported in preclinical studies in recent years [10–12], some of which even demonstrated cross-protection against multiple GPS serotypes in mouse models. Immunoproteomics based on 2-D gel electrophoresis has also been used to identify novel candidate vaccine antigens with cross-protective potential against GPS [13]. Meanwhile, a variety of subunit vaccine antigens have been evaluated for protective effects in laboratory. These antigens include pili subunit PilA [14], transferrin-binding proteins TbpA and TbpB [15–17], trimeric autotransporter VtaA [18], polyamine transport protein PotD [19], lipoprotein VacJ [20, 21], and glyceraldehyde-3-phosphate dehydrogenase GAPDH [22, 23]. However, most of these reported candidates have only been validated in mouse models rather than pigs, the natural host. Use of these antigens alone has been shown to provide only partial protection or limited cross-protection against heterologous GPS infection. Thus, screening highly immunogenic antigens and combining two or more antigens into multicomponent cocktail vaccines is a promising strategy to improve the cross-protective efficacy of GPS subunit vaccines.

In this study, a systematic screening of antigens was performed using in silico immunogenicity, in vivo protective assays, and adjuvant optimization in a murine GPS infection model. A multi-antigen subunit cocktail vaccine was successfully developed. This vaccine exhibited prominent immunogenicity, as it elicited robust humoral and cellular immune responses and conferred effective cross-protection against GPS4 and GPS5 infections in mice. The vaccine also demonstrated favorable immune protective efficacy against GPS infection in piglets in a pilot study. Collectively, these results suggested that the subunit cocktail vaccine exhibits great potential for preventing GPS infection under experimental conditions. However, further large‑scale pig trials and evaluations against more GPS serotypes are still required to validate its broad protective spectrum and practical applicability in field production.

## Materials and methods

### Bacterial strains and growth conditions

All strains used in this study are listed in Additional file 1. *E. coli* strains were cultured in Luria–Bertani (LB; Becton Dickinson) liquid medium or on LB solid agar plates containing ampicillin (100 µg/mL) or kanamycin (50 µg/mL). GPS moderate virulent serotype 4 (GPS4, HP-LH39) and high virulent serotype 5 (GPS5, HP-S15) strains were kindly provided by Prof. Lei Wang (Henan Institute of Science and Technology, Xinxiang, China) [10]. Both strains were isolated from diseased pigs and were cultivated in Brain Heart Infusion (BHI; Hopebio) medium supplemented with 5% horse serum and 20 µg/mL NAD (BHI+) or plated on BHI+ agar plates. All cultures were incubated at 37 °C, 200 rpm with 5% CO_2_ if not specified.

### Antigen selection and DNA manipulation

In this study, a total of 17 GPS candidate antigens, including PotD (GenBank: ACL31786.1), MnSOD (GenBank: ACL32450.1), GPD (GenBank: ACL32465.1), YgiW (GenBank: ACL33049.1), Omp26 (GenBank: ACL32860.1), QseB (GenBank: ACL33513.1), GAPDH (GenBank: ACL31707.1), TEX (GenBank: ACL32154.1), PalA (GenBank: ACL31779.1), ApbE (GenBank: ACL31801.1), PtsG (GenBank: ACL33546.1), HutZ (GenBank: ACL32674.1), KefA (GenBank: ACL32066.1), VacJ (GenBank: ACL33700.1), ClpP (GenBank: ACL33474.1), CRP (GenBank: ACL33503.1), and QueA (GenBank: ACL32317.1), were initially selected for the analysis of the antigenicity using VaxiJen V2.0 [24]. The proteins with good antigenicity (≥ 0.4) were chosen for further analysis. Finally, 14 GPS antigens were selected and the transmembrane regions and the signal peptides of these antigens were predicted using the TMHMM and SignalP, respectively [25, 26]. The protein sequences of these antigens were retrieved from the National Center for Biotechnology Information (NCBI) database. Fragments without transmembrane regions and the signal peptides were amplified by polymerase chain reaction (PCR) from the genomic DNA extracted using a gDNA isolation kit (Qiagen). The PCR products were digested with BamHI/XhoI (NEB) restriction enzymes and ligated into pET28a or pET32a vector using a Rapid DNA ligation kit (Roche Diagnostics). Inserted DNA sequences were confirmed by DNA sequencing and double digestion by BamHI/XhoI (NEB) restriction enzymes. All plasmids and primers used in this study are listed in Additional file 1 and Additional file 2, respectively.

### Protein purification

The constructed plasmids were transformed into *E. coli* BL21 (DE3). When grown to OD_600_ = 0.6–0.8, 0.5 mM of IPTG was added to induce protein expression for 16 h at 16 °C, 180 rpm. Bacterial cells were disrupted by sonication and the supernatants were collected after centrifugation at 12 000 × *g* for 30 min at 4 °C. Protein purification was performed using high-affinity Ni–NTA resin (GeneScript) by gravity at a flow rate of 1 mL/min. The resin was washed with a 5-bed volume of washing buffer (50 mM NaH_2_PO4 pH 8.0, 300 mM NaCl, 20 mM imidazole, 0.1% Triton X-114) to remove endotoxin bound to the resin and the target protein. Proteins were eluted using 500 mM imidazole and dialyzed subsequently against washing buffer using Amicon filter units (Merck; 10-kDa cutoff) at 4 °C. The purity of all recombinant proteins was evaluated by 12% or 15% sodium dodecyl sulfate-polyacrylamide gel electrophoresis (SDS-PAGE) followed by Coomassie brilliant blue staining. Protein concentration was measured by a bicinchoninic acid assay (BCA) kit (Thermo Scientific) and stored in aliquots at −80 °C.

### Prediction of tertiary structures, molecular docking, and immune simulation

The tertiary structures of PtsG, VacJ, PalA, and GAPDH were predicted by AlphaFold2. The binding affinity between each antigen and immune receptors, including TLR2 (PDB ID: 2z7x), MHC II (PDB ID: 5jlz), and swine leukocyte antigen SLA-1 (PDB ID: 3qq3), were assessed by molecular docking using the ClusPro 2.0 server [27]. The docking results were visualized with Pymol and the interaction residues between the docked chains were analyzed using PDBsum [28]. The immune response profile of each antigen was simulated using the C-ImmSim server [29]. The time steps of the three injections in the simulation phase were set to 1, 42, and 84, respectively. The standard time of one injection was 8 h, corresponding to a 14-day gap between immunizations. The simulation volume was set to 50 and the simulation step to 1050, with all other parameters kept at default values.

### Immunization and challenge of mice

For immunization of mice, 210 6-week-old healthy female Institute of Cancer Research (ICR) mice (body weight 18 ± 2 g, purchased from Changsheng Biotechnology Co., Ltd., China) were randomly divided into different groups (10 mice/group). For selection of immunoprotective antigens and antigen combinations, mice were immunized with 100 μg antigens (combinations) mixed with Gel-01 adjuvant (10%; SEPPIC, France), Phosphate-Buffered Saline (PBS) plus Gel-01 (control group), and inactivated vaccine by multipoint subcutaneous injection on the backs of mice, respectively. For selection of optimal adjuvant, mice were immunized with different adjuvants in PBS using the same administration route. Detailed grouping information are listed in Additional file 3. The immunization was administered every 2 weeks for a total of two doses. Blood was collected from the tail vein of mice at 14 and 28 days after first dose to detect specific antibody levels by enzyme-linked immunosorbent assay (ELISA). Alternatively, blood was collected from the tail vein of mice at 0, 2, 4, 6, 8, 9, 10, and 11 weeks after the second dose to monitor the antibody levels by ELISA. After 2 weeks of the second dose, the mice were challenged with lethal dose of GPS4 (4 × 10^9^ CFU, 2 × LD_50_) and GPS5 (2.4 × 10^9^ CFU, 2 × LD_50_) by intraperitoneal injection. Animal death and body weight was recorded every 24 h for 1 week. The mice were euthanized using CO_2_ inhalation or when they met humane endpoints: ≥ 20% lost of maximum body weight for two consecutive days, immobility, or moribund status.

### Immunization and challenge of piglets

In this study, six 4-week-old healthy Landrace piglets (specific pathogen free, purchased from Zybio, China) were housed in a standardized environment (temperature 25 ± 2 °C, relative humidity 50–60%, 12 h light/dark cycle, ad libitum access to sterile feed and clean water) for a 3-day acclimation period prior to the first vaccination. During this period, clinical health symptoms (including rectal temperature, feed intake, mental state, and respiratory status) were assessed to confirm all piglets were free of underlying infection, clinical abnormalities, and transport stress before the formal immunization procedure. For immunization, the piglets were randomly divided into two groups (three piglets/group) and immunized with a total of 1.5 mg antigens mixed with Gel-01 adjuvant (10%; SEPPIC, France) and PBS plus Gel-01 by intramuscular injection in neck, respectively. The immunization was administered every 3 weeks for a total of two doses. Body temperature, mental status, and feeding behaviors of piglets were recorded everyday after the first immunization for 1 week. Blood was collected from anterior vena cava of piglets at 21 and 35 days after first dose to detect specific antibody levels by ELISA. After 2 weeks of the second dose, the piglets were challenged with GPS5 (4 × 10^10^ CFU) by intraperitoneal and intranasal injection. The piglets were examined twice a day for 7 days, and the observations of body temperature, general appearance, nervous signs, and other signs were recorded by a scoring system and summed to give a total clinical score [30]. Piglets showing severe neurological symptoms, inability to stand, anorexia for more than 24 h, severe dyspnea, or rectal temperature above 41.5 °C for more than 48 h would be euthanized immediately to avoid unnecessary suffering. No piglets reached the humane endpoint before the scheduled 7-day observation period in this study. The piglets were sedated with an intramuscular injection of tiletamine-zolazepam (Zoletil), and after confirming that the piglets had completely lost consciousness and nociception, euthanasia was performed by rapid exsanguination via the anterior vena cava.

### ELISA

An indirect ELISA was used to detect the specific antibody levels in animal serum. In brief, the antigens were diluted separately with coating buffer (0.1 mol/L Na_2_CO_3_-NaHCO_3_, pH 9.6) to 1 μg/mL and coated on ELISA plate by 100 μL/well at 4 °C overnight. After washing, 100 μL of 5% bovine serum albumin (BSA) was added to each well and sealed at 37 °C for 1 h. Then, the sera were diluted ten times and added to ELISA plate (100 μL/well). Each serum sample was tested in triplicate for each dilution, and samples with a coefficient of variation (CV) between triplicate wells exceeding 10% were retested. Each immunization group included ten independent biological replicates (n = 10 mice per group). Negative control was set up as the serum from the PBS + Adjuvant immunized group collected at the same time point. Blank control wells were incubated with blocking buffer instead of serum to correct background absorbance. After incubation at 37 °C for 1 h, goat anti-mouse immunoglobulin G (IgG) horseradish peroxidase (HRP; Abclonal, AS003; 1:5000) or goat anti-pig IgG HRP (Solarbio, SE137; 1:5000) was added to each well (100 μL/well) to detect specific antibody levels in the serum of mice and pigs, respectively. After incubation for another 1 h, the reaction was visualized by addition of TMB reagent (100 μL) and terminated by 2 M H_2_SO_4_ (50 μL). Absorbance was detected at OD_450_ nm. The cutoff value for positive antibody judgment was set as 2.1 times the average OD_450_ value of the negative control serum.

### Whole blood killing

GPS4 and GPS5 from the midlog growth phase were collected, centrifuged, washed twice by PBS, and adjusted to 2 × 10^9^ CFU. Bacterial suspensions (10 μL) were mixed with 150 μL of mice blood (collected from individual immunized mice, n = 10) with or without immunization and incubated for 2 h at 37 °C. The mixture was serially diluted and plated on BHI+ agar plates for cell counting. Each blood sample was tested in triplicate. Bacterial survival was calculated as follows: (1 − recovered CFU/CFU in the original inoculum) × 100%. Three independent experiments were performed.

### Western blot

Identification and the antigenicity of the purified proteins VacJ, PtsG, GAPDH, and PalA were assessed by SDS-PAGE and western blot using anti-His antibody and GPS4 or GPS5 antibody-positive serum, respectively. The proteins were boiled in SDS sample buffer at 95 °C for 10 min and then separated on 12.5% SDS-PAGE gels. After electrophoresis, gels were transferred to the PVDF membrane (Millipore) and blocked with 3% BSA overnight. After washing in Tris-buffered saline with Tween‑20 (TBST), the membranes were incubated with rabbit anti-His (1:3000; Abconal, AE068) or GPS antibody-positive serum at room temperature for 2 h. After washing in TBST, the membranes were incubated with goat anti-rabbit HRP-IgG (1:5000; Abconal, AS014) for another 1 h at room temperature. The targeted proteins were visualized using ECL reagent (Millipore) under a luminescent imaging system (Tanon 5200 Multi).

### Flow cytometry

After 2 weeks of the second immunization, the mice were euthanized by inhalation of CO_2_. Mice spleen were collected, cut into pieces, and digested in Hanks’ balanced salt solution (HBSS) containing gelatinase A (100 U/mL) and DNase (20 µg/mL). Following washing with PBS, tissue samples were exposed to ice-cold RBC lysis buffer for 5 min. The cell pellets were gathered by centrifugation and suspended in fluorescent washing buffer (ThermoFisher) to a concentration of 1 × 10^7^/mL. Then, cells were labeled with fluorescent antibodies, including PerCP/Cy5.5 anti-mouse CD45 (Biolegend, 103132), FITC anti-mouse CD3 (Biolegend, 100203), APC anti-mouse CD4 (Biolegend, 100515), PE-Cy7 anti-mouse CD8 (Biolegend, 100722), APC anti-mouse CD19 (Biolegend, 152409) antibodies, and incubated in the dark for 30 min. Moreover, single-stained control tubes were prepared using the same batch of antibodies and fresh mouse spleen single-cell suspension, along with an unstained blank control tube. After washing with PBS, cells were analyzed using a flow cytometer (CytoFLEX). Data were processed using FlowJo software (Version 10.4). A unified gating strategy was supplemented in Additional file 4.

### Histopathological analysis and bacterial loads in organs

The histopathological analysis was performed as described previously [31]. Briefly, lung, liver, and spleen tissues were collected 1 week after GPS challenge in mice and piglets. The tissues were fixed with 4% paraformaldehyde solution. After dehydration via graded ethanol, the tissues were cleared with xylene, embedded in paraffin, and sliced (3–5 μm) using a microtome. Then, the slices were mounted on slides, deparaffinized, rehydrated, and stained with hematoxylin–eosin (HE). After dehydration, slides were coverslipped for microscopic examination. To analysis bacterial loads in organs, equal weights of lung, liver, spleen, and blood samples were collected from individual immunized mice (n = 10) 1 week after GPS challenge in mice and piglets and homogenized with PBS (1 g/3 mL). Then, the samples were serial diluted and spotted on BHI+ agar plates in triplicate. The number of viable bacteria were counted after overnight incubation at 37 °C. Three independent experiments were performed.

### Lymphocyte proliferation assay

After 2 weeks of the second immunization, the ICR mice were euthanized by cervical dislocation and the spleens were aseptically harvested from each mice (n = 10). Single-cell suspensions were prepared after red blood cell lysis. Cells were resuspended in complete RPMI 1640 medium containing 10% inactivated fetal bovine serum and 1% penicillin–streptomycin. Isolated lymphocytes were seeded in a 96-well plate at a density of 5 × 10^6^ and treated with either PBS or VA+PT+GA (10 μg/well, 1:1:1). After incubation at 37 °C for 48 h, cell viability was assessed using a CCK-8 kit (Beyotime, China) according to the manufacturer’s instructions. Each sample was set up in triplicate wells. Three independent experiments were performed. Absorbance was detected at 450 nm. Alternatively, total RNA was isolated from different groups of cells from a 6-well plate under a same procedure of stimulation.

### RNA isolation and quantitative real-time PCR

Total RNA from individual mouse tissues (n = 10) was extracted using a tissue RNA isolation kit according to the manufacturer’s instructions (Invitrogen, USA). The extracted RNA was quantified using a Nanodrop 2000 spectrophotometer (Thermo Scientific) and its quality was assessed by PCR and gel electrophoresis. cDNA is synthesized from 1 μg of purified RNA using Prime Scrip RT Master Mix Kit (TaKaRa, China). Each cDNA sample was amplified in triplicate. Quantitative PCR was then performed with gene-specific primers using TB Green PCR Master Mix (TaKaRa, China) on a real-time PCR system (q225, Kubo Instruments, China). Data were analyzed using the 2^−ΔΔCT^ method. Amplification curves are analyzed to determine cycle threshold (Ct) values, with target gene expression normalized to reference gene (*GAPDH*) for relative quantification. Three independent experiments were performed. Primers are listed in Additional file 2.

### Statistical analysis

The experimental data were statistically analyzed using GraphPad Prism (version 9.0). Data are presented as mean ± SD from three independent replicates. Differences between the mean values of normally distributed data were assessed using one-way and two-way analysis of variance (ANOVA; Dunnett’s test). A *p*-value < 0.05 was considered to be significant and indicated by “*”. A *p*-value < 0.01 was considered to be extremely significant and indicated by “**”.

## Results

### Selection of GPS candidate antigens

**Figure 1:**
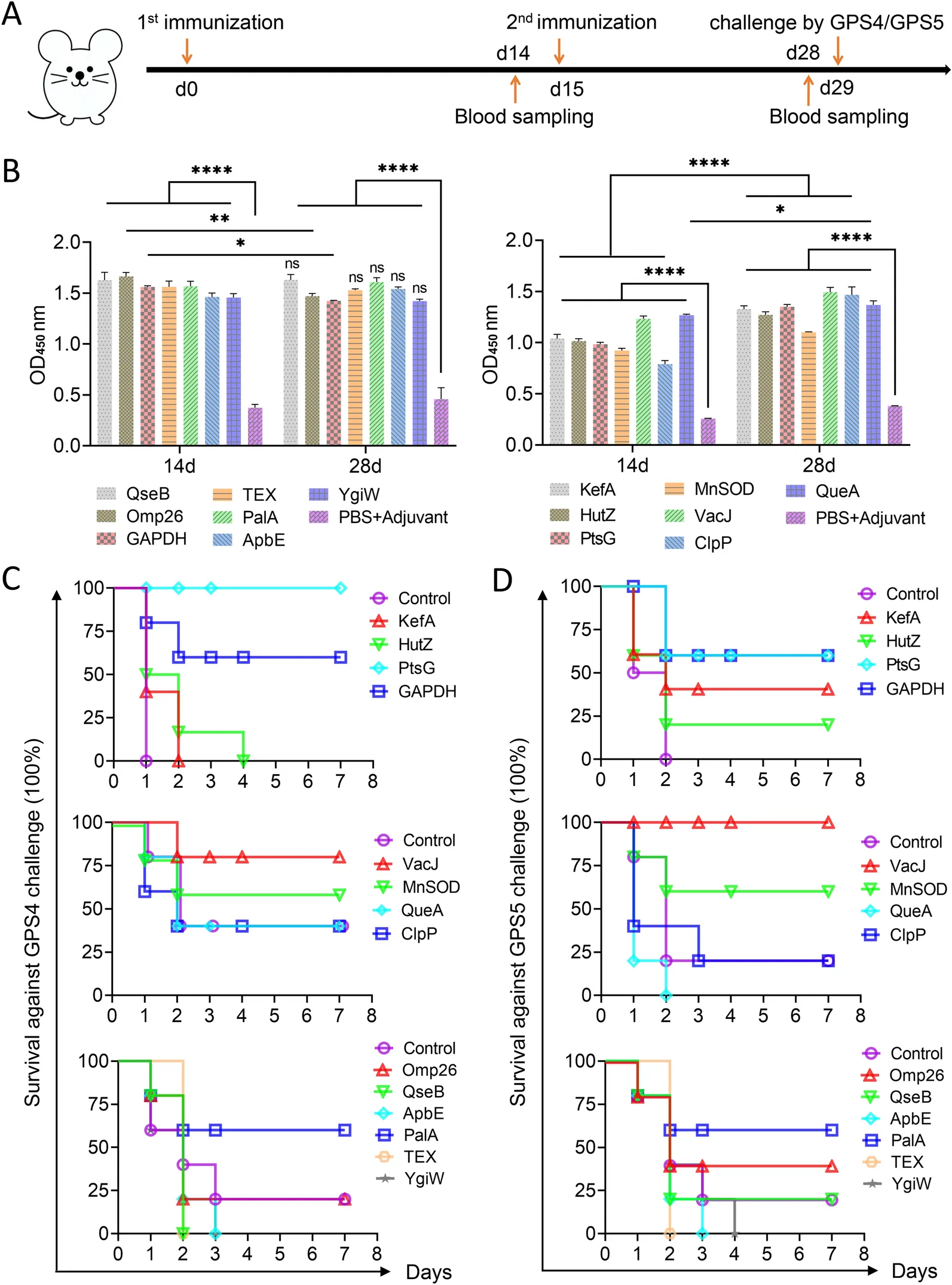
**Selection of the antigens with high immunoprotective efficacy in mice.** (**A**) Schematic representation of the immunization program of mice. (**B**) Detection of the production of antigen-specific antibodies in mice serum at 14 and 28 days after immunization by ELISA. Different groups of mice (n = 10) were immunized with 100 μg antigens mixed with 10% Gel-01 adjuvant by multipoint subcutaneous injection. The immunization was administered every 2 weeks for a total of two doses. 14 days, 14 days post-immunization; 28 days, 28 days post-immunization. (**C**, **D**) Survival curve of immunized mice upon challenge by GPS4 (**C**) and GPS5 (**D**). Mice were challenged with GPS4 (4 × 10^9^ CFU) and GPS5 (2.4 × 10^9^ CFU) by intraperitoneal injection after 2 weeks of the second dose of immunization. Animal death was recorded every 24 h for 1 week. Data were expressed as mean ± SD and analyzed using two-way ANOVA (**B**). ns, not significant, *p < 0.05, **p < 0.01, ***p < 0.001, and ****p < 0.0001.

To identify antigens for an effective subunit vaccine for GPS infection, we first selected 17 potential or characterized antigens of GPS and evaluated their antigenicity using the VaxiJen V2.0 server at threshold of 0.4. Except PotD, GPD, and CRP, all other 14 antigens retrieved scores higher than 0.4 (Additional file 5). These 14 antigens were thus chosen for further analysis. Next, the coding sequences of these antigens were cloned in either pET28a or pET32a expression vectors for protein expression (Additional file 6A). The expression profiles of the His-tagged recombinant proteins were analyzed by SDS-PAGE; 13 of the 14 recombinant proteins showed clear single band with hardly visible nonspecific impurity bands or target protein degradation in the SDS-PAGE gel (Additional file 6B), suggesting these proteins were successfully purified with high purity. However, one protein (KefA) showed distinct nonspecific impurity bands even after multiple rounds of purification optimization. Subsequently, the immunoprotective effect of the recombinant proteins were preliminarily evaluated in a murine infection model. Mice were subjected to a two-step immunization regimen (100 μg of each antigen by multipoint subcutaneous injection on the backs of the mice), followed by challenge with GPS4 and GPS5 at 14 days after the secondary immunization (Figure 1A). The results showed that the levels of antigen-specific antibodies in the mice’s serum were significantly increased after immunization at 14 and 28 days compared with the control group (PBS+Adjuvant) (Figure 1B). Notably, half of the antigens induced significant higher antibody levels after the second immunization than those after the first immunization. These findings indicate that these antigens have good immunogenicity that can trigger a strong humoral immune response after immunization in mice. Moreover, immunization of mice with PtsG, GAPDH, VacJ, and PalA increased mice survival rate to 100%, 60%, 80%, and 60%, respectively, upon infection with GPS4 compared with control group (0%, 0%, 35%, 20%, correspondingly), while the survival rates for the immunization with other antigens were all below 60% (Figure 1C). Upon infection with GPS5, immunization of mice with PtsG, GAPDH, VacJ, MnSOD, and PalA increased mice survival rate to 60%, 60%, 100%, 60%, and 60%, respectively, compared with control group (0%, 0%, 20%, 20%, 20%, correspondingly), while the survival rates for the immunization with other antigens were all below 40% (Figure 1D). Collectively, immunization with PtsG, GAPDH, VacJ, and PalA conferred to better immunoprotective effect than other antigens in mice against both GPS4 and GPS5 infection.

Furthermore, the tertiary structures of PtsG, GAPDH, VacJ, and PalA were predicted and their binding affinities with host immune receptors TLR2, MHC II, and swine leukocyte antigen (SLA I) were docked to assess the capability to induce immune response. Molecular docking results demonstrated that all the four antigens exhibited good binding affinity to both murine and porcine immune receptors (Additional file 7), suggesting they may have a strong capability to activate innate immune cells. Correspondingly, a broad and robust activation of the immune system post-injection was observed by immune simulation using the C-ImmSim server (Additional file 8). Simulated immunization of these antigens not only increased the total number of T cells and B cells, but also upregulated cytokine responses and the level of antibodies, indicating these antigens are capable of inducing strong immune responses in silico. Notably, detection of the antigenicity of the purified proteins illustrated that these antigens can be recognized by both GPS4- and GPS5-positive antiserums (Additional file 9), suggesting that they could possibly confer to cross-protection against both GPS4 and GPS5 infection in mice. Based on the observations above, these four antigens were chose for the construction of cocktail vaccine.

### Development and optimization of antigens for cocktail vaccine

**Figure 2:**
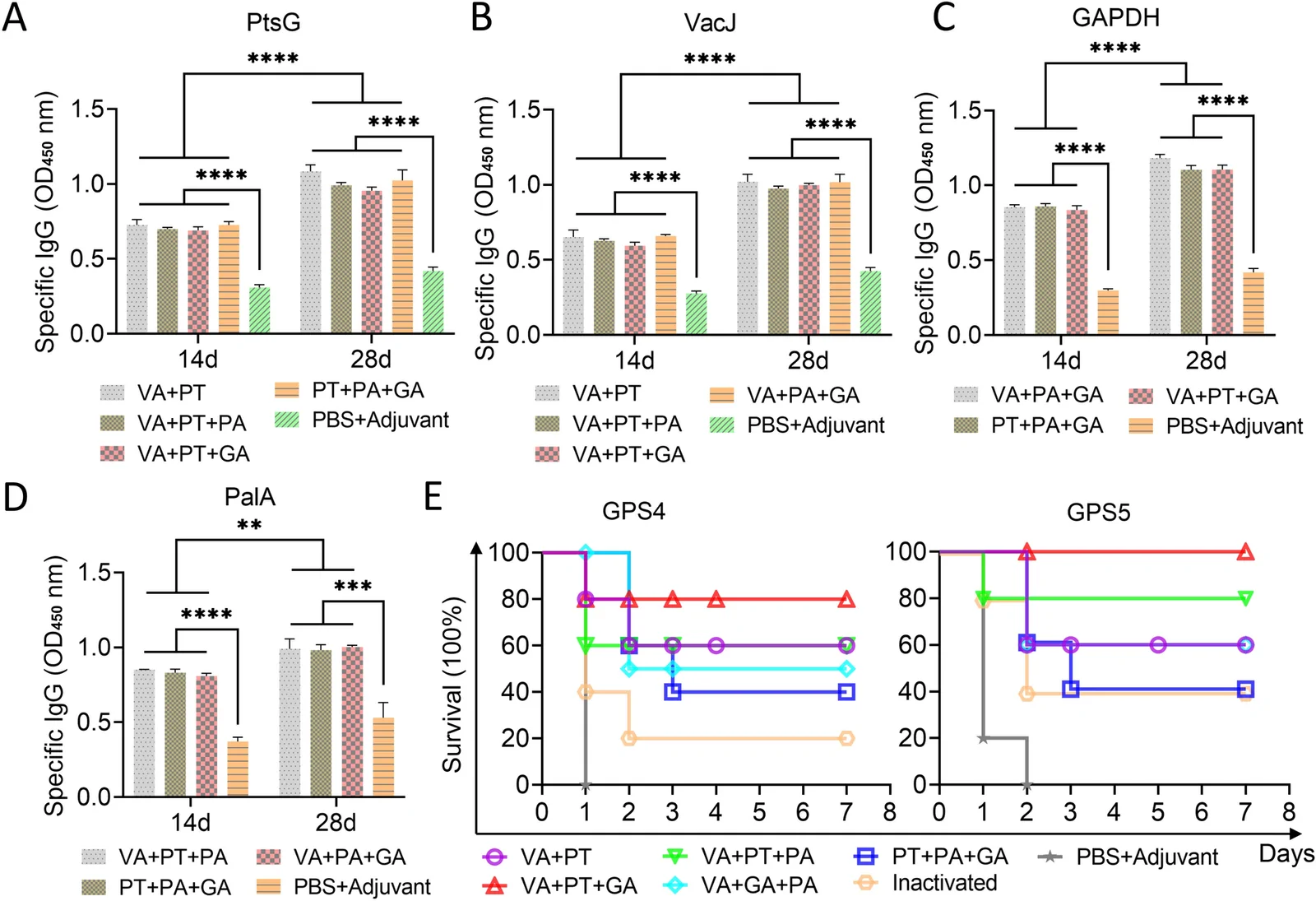
**Screening of optimal antigen combination for the cocktail vaccine.** (**A**–**D**) Detection of the production of antibodies specific to PtsG (**A**), VacJ (**B**), GAPDH (**C**), PalA (**D**) in mice serum at 14 and 28 days after immunization by ELISA. Different groups of mice (n = 10) were immunized with a total of 100 μg antigen combinations mixed with 10% Gel-01 adjuvant by multipoint subcutaneous injection. The immunization was administered every 2 weeks for a total of two doses. 14 days, 14 days post-immunization; 28 days, 28 days post-immunization. (**E**) Survival curve of mice upon challenge with GPS4 (4 × 10^9^ CFU) and GPS5 (2.4 × 10^9^ CFU) by intraperitoneal injection after 2 weeks of the second dose of immunization. Animal death was recorded every 24 h for one week. VA+PT, VacJ+PtsG; VA+PT+PA, VacJ+PtsG+PalA; VA+PT+GA, VacJ+PtsG+GAPDH; PT+PA+GA, PtsG+PalA+GAPDH; VA+GA+PA, VacJ+GAPDH+PalA. Data were expressed as mean ± SD and analyzed using two-way ANOVA (**A**–**D**). ns, not significant, *p < 0.05, **p < 0.01, ***p < 0.001, and ****p < 0.0001.

To further improve the immunoprotective effect of the antigens, we randomly combined two or three antigens into different groups and immunized mice with Gel-01 adjuvant. As shown in Figure 2A–D, the levels of specific antibodies in the mice’s serum were significantly increased after immunization at both 14 and 28 days compared with the control group. Of note, PalA-specific antibody levels increased after the second immunization, but to a lesser extent than those for the other three antigens (Figure 2D). Furthermore, compared with the control group, immunization regimens including VA+PT (VacJ and PtsG), VA+PT+GA (VacJ, PtsG and GAPDH), and VA+PT+PA (VacJ, PtsG and PalA) all conferred more than 50% protection in mice following GPS4 challenge, with the VA+PT+GA exhibiting the optimal efficacy at 80% (Figure 2E). Similarly, all the above groups provided more than 60% protection after GPS5 challenge, among which VA+PT+GA achieved complete protection (100%) (Figure 2E). Notably, the protective efficacy of VA+PT+GA immunization was significantly superior to that of the commercial inactivated vaccine group, which only conferred 20% and 40% protection against GPS4 and GPS5 challenges, respectively (Figure 2E). These results indicate that VacJ, PtsG, and GAPDH were the optimal antigen combination for the cocktail vaccine.

### Screening of optimal immune adjuvants

**Figure 3:**
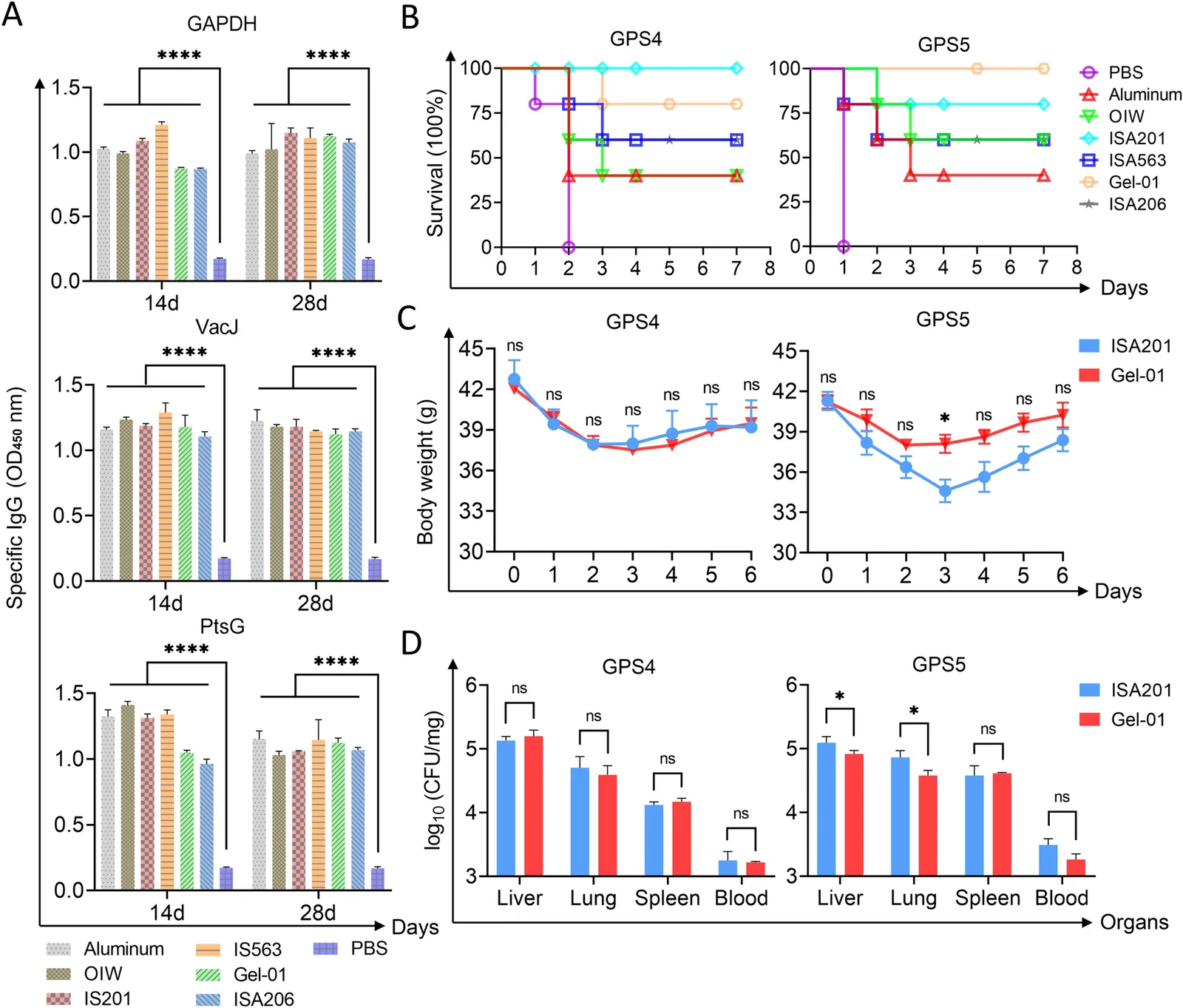
**Screening of optimal immune adjuvants.** **A** Detection of the production of antigen-specific antibodies in mice serum at 14 and 28 days after immunization by ELISA. Mice (n = 10) were immunized with a total of 100 μg antigens (PtsG:VacJ:GAPDH = 1:1:1) mixed with different adjuvants including Montanide™ ISA201, ISA206, ISA563, Gel-01, aluminum hydroxide (Aluminum), and white oil (OIW). The immunization was administered every 2 weeks for a total of two doses. 14 days, 14 days post-immunization; 28 days, 28 days post-immunization. (**B–C**) Survival curve (**B**) and body weight changes (**C**) of immunized mice after challenge with GPS4 (4 × 10^9^ CFU) and GPS5 (2.4 × 10^9^ CFU) by intraperitoneal injection. Animal death and body weight was recorded every 24 h for one week. (**D**) Assessment of bacterial loads in key organs of immunized mice after GPS4 and GPS5 challenge. Data were expressed as mean ± SD and analyzed using two-way ANOVA (**A**, **C**, **D**). ns, not significant, *p < 0.05, **p < 0.01, ***p < 0.001, and ****p < 0.0001.

Adjuvants are critical in improving vaccine efficacy, so we further screened the optimal adjuvants to identify those that best enhance the immunogenicity and protective efficacy of the cocktail vaccine. To this end, antigen combination of VacJ, PtsG, and GAPDH was emulsified with a number of commonly used adjuvants, including Montanide™ ISA201, ISA206, ISA563, Gel-01, aluminum hydroxide, and white oil (OIW), and immunized mice. In terms of antibody responses, all adjuvant groups exhibited significantly higher antibody levels than the control group, regardless of primary or secondary immunization (Figure 3A). Following the primary immunization, the traditional aluminum hydroxide adjuvant group induced the highest titers of GAPDH- and VacJ-specific antibodies compared with other adjuvant groups; however, these antibody levels declined after the secondary immunization, with a similar trend observed in the induction of PtsG-specific antibodies. In contrast, although the ISA201 and Gel-01 adjuvant groups did not elicit the highest antibody levels relative to other adjuvant groups, they showed increased GAPDH- and PtsG-specific antibody titers after the secondary immunization. Moreover, across all adjuvant-immunized groups, a certain level of protective efficacy against both GPS4 and GPS5 challenges was observed in mice. Notably, the ISA201 and Gel-01 adjuvant groups exhibited the most robust protective effects, with survival rates of 100% and 80%, respectively, following GPS4 challenge, and 80% and 100%, respectively, upon GPS5 challenge (Figure 3B). Analysis of murine body weight changes revealed no significant difference in weight changes between the two groups following GPS4 challenge. However, after GPS5 challenge, mice in the ISA201 adjuvant group exhibited significantly more pronounced weight changes compared with those in the Gel-01 adjuvant group (Figure 3C). Consistently, mice immunized with Gel-01 exhibited lower bacterial loads in the lung and liver compared with those in the ISA201 adjuvant group upon GPS5 challenge (Figure 3D). Overall, these results indicate that Gel-01 was the optimal adjuvant of the cocktail vaccine.

### Immunization of the cocktail vaccine enhanced both cellular and humoral immune responses

**Figure 4:**
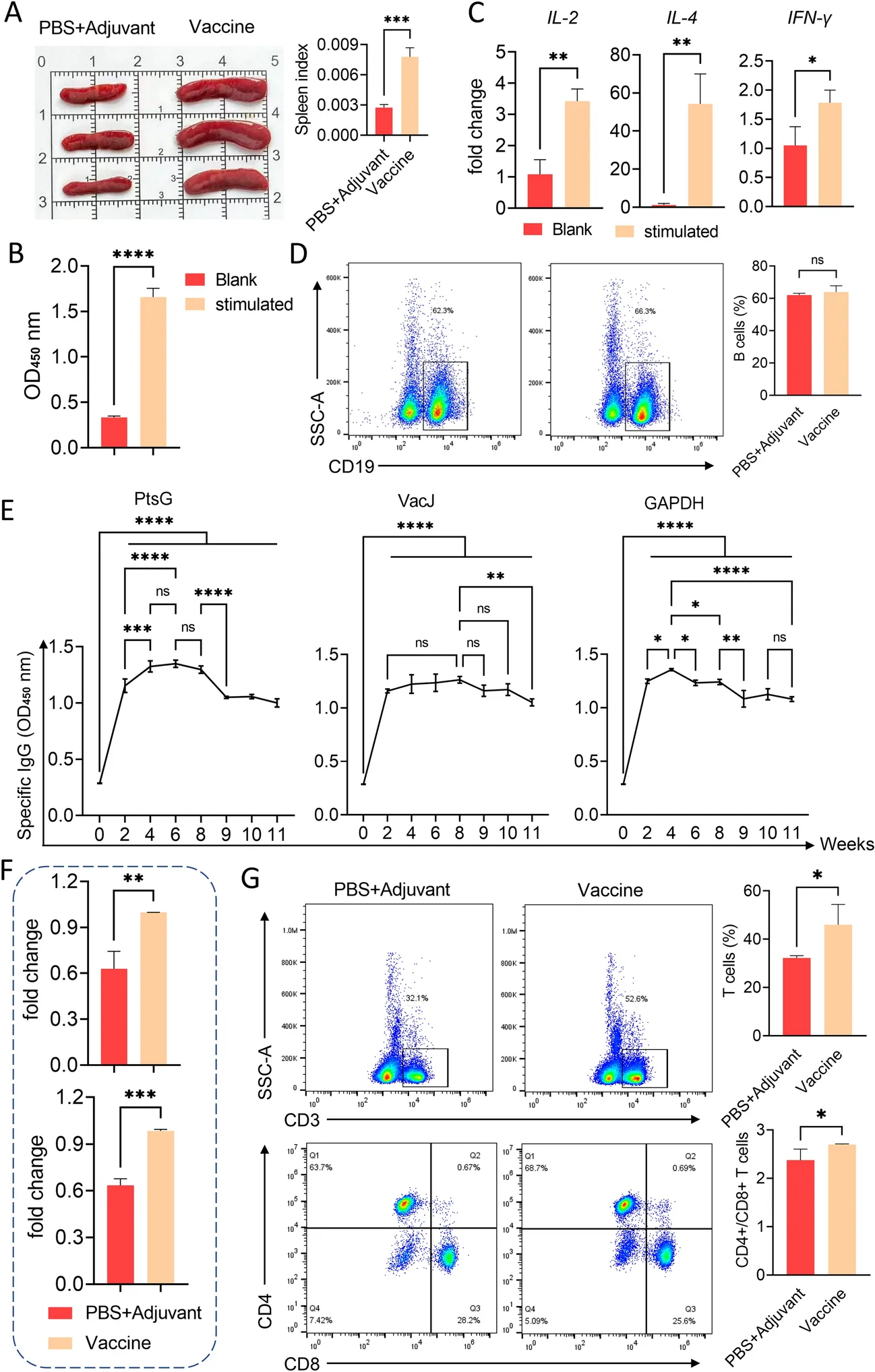
**Evaluation of host cellular and humoral immune responses following immunization of the cocktail vaccine in mice.** **A** Assessment of spleen index at 28 days after immunization. Spleen index = spleen weight/body weight. Three representative photographs are shown. (**B**, **C**) Measurement of lymphocyte proliferation by CCK-8 assay (**B**) and the transcriptional levels of *IL-2*, *IL-4*, and *IFN-γ* by qRT-PCR (**C**) after incubation with VA+PT+GA (10 μg, 1:1:1) for 48 h in isolated splenocytes after immunization. (**D**) Analysis of B cells by flow cytometry at 28 days after immunization. (**E**) Detection of the production of antigen-specific antibodies in mice serum by ELISA. The antibody levels were monitored at 0, 2, 4, 6, 8, 9, 10, 11 weeks after the second immunization. (**F**) Bactericidal analysis of the antigen-specific antibodies against GPS4 (above) and GPS5 (below). (**G**) Analysis of the proportion of CD3^+^, CD3^+^CD4^+^ and CD3^+^CD8^+^ T cells by flow cytometry. Data were expressed as mean ± SD and analyzed using unpaired *t*-test (**A**–**D**, **F**, **G**) and one-way ANOVA (**E**). ns, not significant, *p < 0.05, **p < 0.01, ***p < 0.001, and ****p < 0.0001.

Next, the effect of the cocktail vaccine on host cellular and humoral immune responses was evaluated. We observed that the splenic index in mice was significantly increased following vaccine immunization compared with the control group (Figure 4A). Splenic lymphocytes were isolated from mice, and stimulation with VA+PT+GA was found to not only significantly enhance lymphocyte proliferation, but also upregulate the transcriptional levels of cytokine genes *IL-2*, *IL-4*, and *IFN-γ* (Figure 4B–C). Considering the roles of these cytokines in lymphocyte differentiation and antibody production, we subsequently analyzed the proportion of B and T lymphocytes in the spleen and antibody titers in the peripheral blood post-immunization. Flow cytometric analysis revealed no significant increase in the overall B cell population in immunized mice relative to controls (Figure 4D); however, serum antibody levels exhibited a highly significant elevation as early as 2 weeks post-immunization, gradually peaking between 4 and 6 weeks (Figure 4E). This result directly clarifies that the increase in antigen-specific antibody levels induced by vaccination is not necessarily dependent on changes in the total number of B cells in the spleen, but on the vaccine antigen-specific B cells. Subsequent to this peak, a gradual decline was observed; however, antibody levels remained substantially elevated, with such high titers persisting for a minimum of 11 weeks. Furthermore, results from the whole-blood bactericidal assay revealed that serum treatment from the vaccine-immunized group significantly reduced the colony counts of both GPS4 and GPS5 (Figure 4F), indicating that the antibodies elicited by the cocktail vaccine exhibited bactericidal activity against GPS4 and GPS5. In terms of T cells, flow cytometric analysis showed that vaccine immunization induced not only a significant increase in the total number of T cells, but also a higher ratio of CD3^+^CD4^+^/CD3^+^CD8^+^ T cells (Figure 4G), suggesting a possible Th2 type immune response elicited by vaccine. Collectively, these findings confirmed the vaccine’s capacity to induce sustained humoral and cellular immunity in response to vaccine immunization.

### Cocktail vaccination reduced pathological lesions and alleviated inflammatory responses in mice

**Figure 5:**
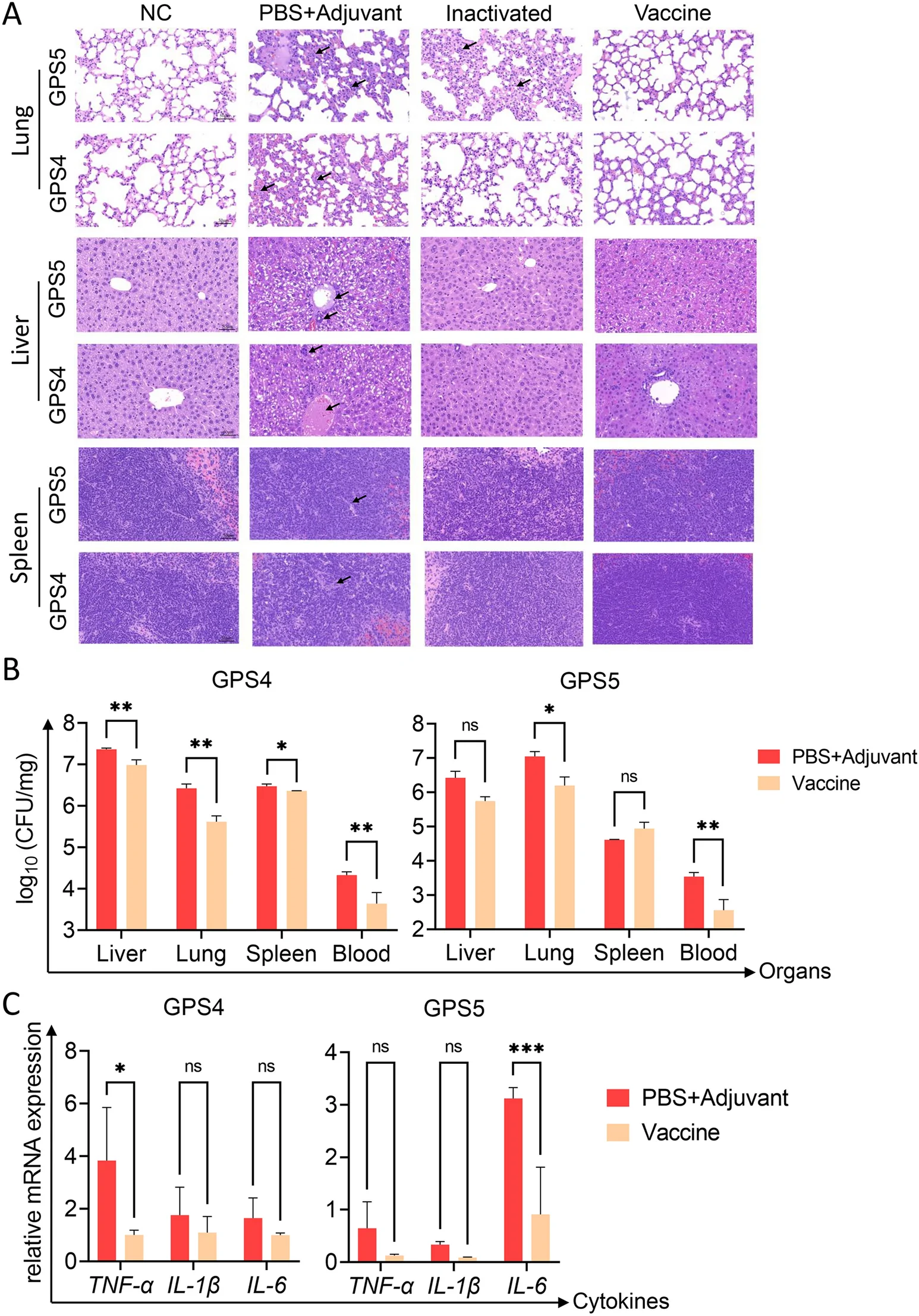
**Assessment of pathological lesions and inflammatory responses in immunized mice following GPS challenge.** (**A**) Histopathological examination of spleen, lung, and liver of mice with or without immunization. NC, immunized with PBS plus adjuvant Gel-01, no GPS challenge; PBS+Adjuvant, challenged by GPS4 or GPS5 after immunization with PBS plus adjuvant Gel-01; vaccine, challenged by GPS4 or GPS5 after immunization with the cocktail vaccine; inactivated, challenged by GPS4 or GPS5 after immunization by commercial inactivated vaccine. Histopathologic features were indicated by black arrows. (**B**, **C**) Assessment of bacterial loads in the spleen, lung, liver, and blood (**B**) and the transcription levels of proinflammatory cytokines in lung tissue (**C**) of mice immunized with the cocktail vaccine following challenge with GPS4 and GPS5. PBS+Adjuvant, challenged by GPS4 or GPS5 after immunization with PBS plus adjuvant Gel-01; vaccine, challenged by GPS4 or GPS5 after immunization with the cocktail vaccine. Data were represented as fold change (**C**). Data were expressed as mean ± SD and analyzed using unpaired *t*-test (**B**, **C**). ns, not significant, *p < 0.05, **p < 0.01, ***p < 0.001, and ****p < 0.0001.

Major organs including liver, lungs, and spleen of mice post-challenge were collected for histopathological analysis. Gross necropsy examination showed that mice challenged with GPS4 or GPS5 exhibited significantly swollen lungs with extensive hemorrhage, congestion, and reddish-brown inflammatory foci compared with the control group (PBS+Adjuvant) (Additional file 10). The lungs in the inactivated vaccine group displayed mild enlargement and partial reddish-brown inflammatory foci, whereas only a few petechiae and congestion spots were observed in the cocktail vaccine-immunized group, with no lung swelling. Similar findings were noted in the spleen and liver of the mice. Histopathological examination showed that being challenged with GPS led to drastic pathological changes in the lungs of the mice, including large amounts of inflammatory exudate, thickening and collapse of alveolar walls, dramatic atrophy of alveoli, and extensive infiltration of red blood cells and inflammatory cells (Figure 5A). Of note, immunization with the inactivated vaccine alleviated the pathological changes to a certain degree, and the cocktail vaccine-immunized group dramatically reduced histopathological lesions, with only limited red blood cell infiltration and focal alveolar wall thickening. Comparable pathological profiles were observed in the spleen and liver of the mice. Analysis of bacterial loads in these organs and blood revealed that immunized with the cocktail vaccine significantly reduced the total number of both GPS4 and GPS5 in lungs and blood, but not spleen and liver compared with the control group (Figure 5B). Moreover, the transcription levels of proinflammatory cytokine *TNF-α*, but not *IL-1β* and *IL-6*, was significantly reduced in lung by immunization with the cocktail vaccine after GPS4 challenge compared with the control group, while the transcriptional level of *IL-6*, but not *TNF-α* and *IL-1β*, was significantly reduced following GPS5 challenge (Figure 5C). Taken together, these results demonstrated that the cocktail vaccine can effectively protect mice against GPS infection by reducing organ lesions and alleviating inflammatory responses.

### Cocktail vaccination protected piglets from GPS infection in a pilot study

**Figure 6:**
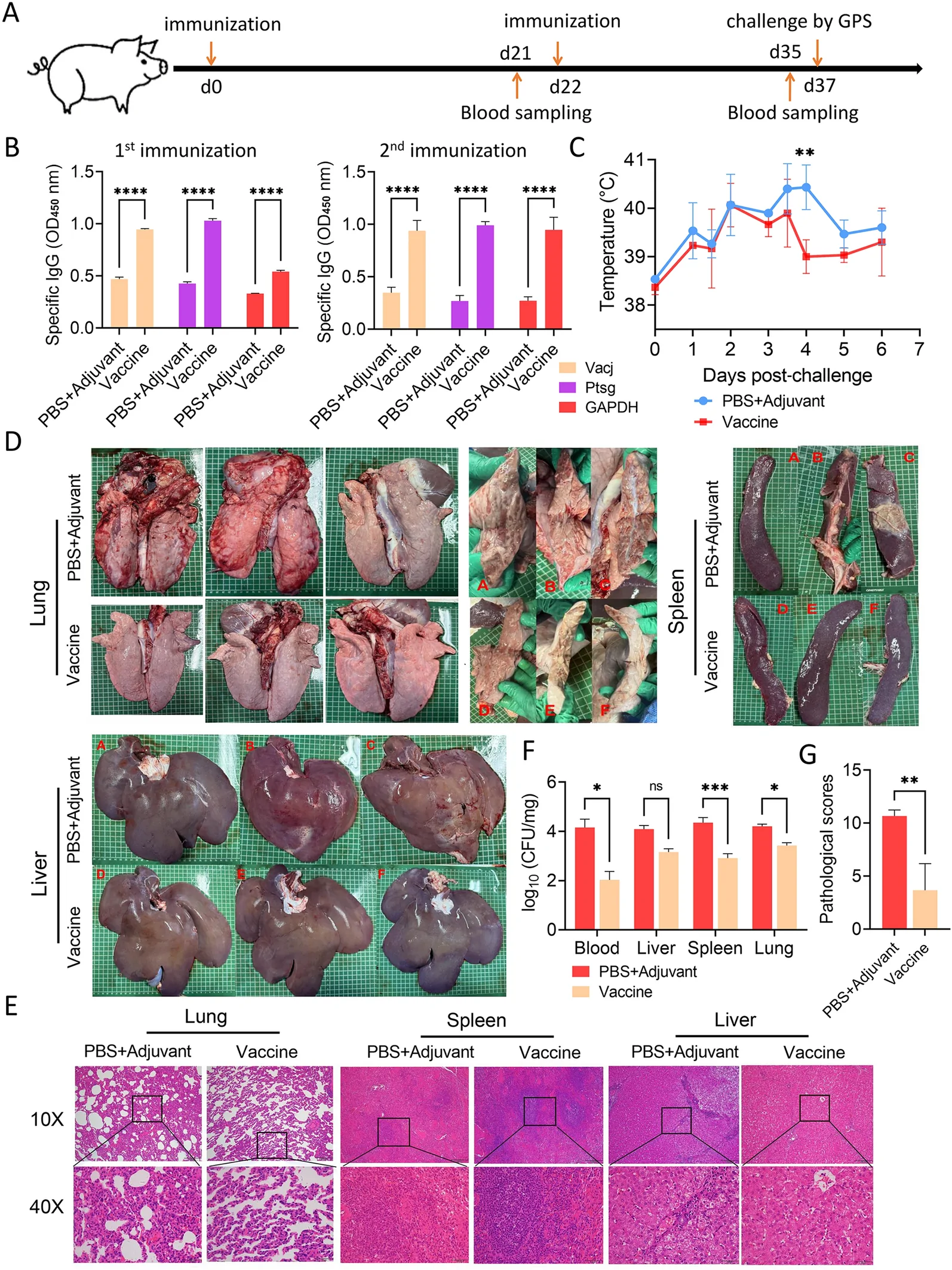
**Assessment of the immunoprotective effect of the cocktail vaccine in piglets.** (**A**) Schematic representation of the immunization program of piglets. (**B**) Detection of antigen-specific antibodies in piglets serum at day 21 (d21) and 35 (d35) after immunization by ELISA. Different groups of piglets (n = 3) were immunized with 1.5 mg antigens mixed with 10% Gel-01 adjuvant by intramuscular injection in neck. (**C**) Body temperature of piglets upon challenge by GPS5 (4 × 10^10^ CFU) via intraperitoneal and intranasal injection after 2 weeks of the second dose. Animal death and clinical symptoms were recorded every 24 h for one week. (**D**, **E**) Gross necropsy (**D**) and histopathological examination (**E**) of spleen, lung, and liver of piglets with or without immunization. The organs were observed on a graduated board (side length of the small squares: 1 cm) and representative images are shown. The red letters in the sub-panels refer to the tissue number of each piglet. (**F**) Assessment of bacterial loads in spleen, lung, liver, and blood of piglets after GPS5 challenge. (**G**) Clinical, lesion, and bacteriological scores of vaccinated and control piglets challenged with GPS5. Data were expressed as mean ± SD and analyzed using two-way ANOVA (**B**, **C**, **F**) and unpaired *t*-test (**G**). ns, not significant, *p < 0.05, **p < 0.01, ***p < 0.001, and ****p < 0.0001.

Given that pigs constitute the target animals for GPS infection, subsequent investigations were conducted to assess the immunoprotective efficacy of the cocktail vaccine in piglets (Figure 6A). Vaccination was delivered via cervical intramuscular injection. Within 24 h post-vaccination, a transient elevation in body temperature was observed in the vaccinated piglets, yet this increase was not statistically significant compared with the control group (Additional file 11). After 24 h, the body temperature of the vaccinated piglets gradually decreased, eventually converging with that of the control group piglets. In terms of antibody responses, high levels of antigen-specific antibodies were elicited following the primary immunization, with titers significantly higher than those in the control group (Figure 6B). After the secondary immunization, although no significant changes were observed in the antibody levels against VacJ and PtsG, the antibody titer against GAPDH increased notably compared with that of the first immunization. These results demonstrated that the cocktail vaccine can induce a favorable humoral immune response in piglets under our experimental conditions.

Two weeks after the second immunization, piglets were challenged with GPS5 and clinical symptoms were observed for 1 week. Without vaccination, GPS5 challenge resulted in a dramatic increase in body temperature; the highest reached 40.2 °C after 4 days post-challenge (Additional file 12; Figure 6C). Moreover, the piglets were accompanied by heavy breathing, nasal discharge, depression, and a tendency to lie down. Piglets in the immunized group also showed elevated body temperature; the highest reached 40 °C after 2 days post-challenge, and then reduced and remained stable thereafter (Additional file 12; Figure 6C). The piglets also exhibited breathing difficulties, nasal discharge, and depression after challenge; however, these symptoms gradually disappeared after 2 days. Notably, the body temperature of the immunized group was significantly lower than that of the control group at 4 days after challenge (Figure 6C). Gross pathological observations following GPS5 challenge revealed that piglets in the control group exhibited substantial fibrinous exudation within the thorax, accompanied by severe adhesions among the heart, lungs, and thoracic wall, which were typical characteristics of fibrinous pleuritis (Additional file 13). The lungs were characterized by enlargement and hemorrhage, with a pale yellow membranous fibrinous exudate present on their surface; well-demarcated congested or hemorrhagic regions were detected in the anterior-inferior segments of the apical, cardiac, and diaphragmatic lobes, as well as in the accessory lobe of both lungs (Additional file 13; Figure 6D). Cross-sectional examination of the lungs demonstrated heterogeneous pigmentation and multiple necrotic foci within the lesioned areas. Similar pathological changes were also observed in liver and spleen. In contrast, the immunized group showed significantly attenuated overall pathological changes compared with the control group upon necropsy. No overt features of pleurisy or peritonitis were noted, and either an absence or only a minimal quantity of fibrinous exudate was observed on the lung surface or within the abdominal cavity. Additionally, there were no significant adhesions between the visceral organs and the thoracic or abdominal walls. Consistent with these findings, the HE-stained sections further showed that GPS5 challenge induced widened alveolar interstitium, alveolar atrophy, and presence of inflammatory exudate in the lungs; the boundary between the red and white pulp in the spleen was obscure with infiltration of inflammatory cells; hepatocytes were swollen and lost their cord-like arrangement in the liver (Figure 6E). In contrast, these pathological manifestations were markedly alleviated in the immunized group. Moreover, although there was no significant difference in the bacterial load in the liver, vaccine immunization significantly reduced the bacterial loads in the blood, lungs, and spleen of piglets compared with the control group (Figure 6F). Finally, a scoring system was used to quantitatively compare the clinical symptoms and pathological damage between groups. The result showed that the scores of immunized group were significantly lower than that of the control group (Figure 6G; Additional file 14).

## Discussion

Porcine Glässer’s disease, an infectious disease caused by GPS, poses a severe threat to swine health due to its high mortality rate, and incurs substantial economic losses in the global swine industry. Vaccination currently stands as the most effective preventive measure against this disease. Nevertheless, existing vaccines are commonly plagued by critical limitations, including short protective durations and poor cross-protection efficacy across different GPS serotypes [1, 4]. In this study, we successfully developed an effective subunit cocktail vaccine against porcine Glässer’s disease. Immunization with this vaccine not only conferred effective cross-protection against GPS4 and GPS5 infections in mice, but also demonstrated favorable immune protective efficacy against GPS infection in piglets in a pilot study, suggesting it may be used as a vaccine candidate against porcine Glasser’s disease in the future.

GPS possesses dozens of virulence factors, many of which are membrane-associated or secreted antigens and are frequently regarded as the antigen candidates for subunit vaccine development. Among the 17 GPS antigens selected in this study, a subset comprises well-characterized GPS virulence factors, including OMP26, YgiW, VacJ, PalA, GAPDH, and MnSOD. Cumulative evidence from relevant studies has demonstrated that immunization with recombinant forms of these antigens elicited substantial immunoprotection across diverse animal models. For example, in a guinea pig model, recombinant VacJ and OMP26 conferred 67% and 83% protection against infection with GPS5 strain SH0165, respectively [20]. In murine models, recombinant GAPDH and PalA achieved protective rates of 75% and 80% against SH0165 challenge, respectively [23, 32]. Notably, previous studies have reported that recombinant MnSOD and YgiW also exhibited cross-protective activity in mice [33, 34]. However, the cross-protective efficacy of these two recombinant proteins was suboptimal (both below 60%) in this study (Figure 1C and D). In addition, it has been demonstrated that immunization of recombinant ApbE combined with two GPS antigens, LpoA and YaeT, exerted limited protection compared with sham vaccinated animals [13], suggesting the antigenicity of ApbE may be weak in piglets.

Besides well-characterized virulence factors, we also incorporated virulence factors with uncharacterized immunogenicity, including PtsG, ClpP, and QseB, as well as the proteins that are significantly upregulated during GPS infection, including Tex, HutZ, KefA, and QueA, all of which have been proposed as potential candidate antigens for GPS vaccine development [13, 35]. Unexpectedly, only recombinant PtsG displayed robust cross-protective efficacy in murine models, whereas the immunoprotective efficacy of the remaining proteins was consistently below 60%. PtsG is a vital protein in the bacterial glucose phosphotransferase system (PTS^Glc^) and mainly in charge of glucose transport as part of the enzyme II complex. PTS^Glc^ not only transports glucose, but also regulates various cellular activities including virulence, biofilm formation, and metabolism [36–38]. For example, deletion of *ptsG* in *Borrelia burgdorferi* was unable to cause infection in mice [39]. PtsG as well as other genes involved in glycolysis are required for the replication of *Salmonella* typhimurium in macrophages within the Salmonella-containing vacuole (SCV) [40]. In GPS, deletion of *ptsG* led to dramatic changes in metabolic and transcriptomic pathways, some of which were associated with bacterial virulence and biofilm formation [36]. However, conclusive experimental evidence addressing whether PtsG regulates the virulence of GPS and whether it qualifies as a antigen for GPS subunit vaccine remains elusive to date. In the present study, we demonstrated, for the first time, that PtsG possessed robust immunogenicity. Immunization of mice with recombinant PtsG alone or in combination with VacJ and GAPDH conferred effective protection against GPS challenge, thereby identifying PtsG as a promising candidate antigen for GPS vaccine development.

Single-antigen vaccines often deliver suboptimal cross-protection against multiple serotypes of pathogenic bacteria. To address this key limitation, combining two or more protective antigens represents an effective approach to enhance the breadth and potency of vaccine protective efficacy [41]. Many studies have confirmed that multi-antigen combination vaccines outperform single-antigen formulations for infectious disease prevention. For example, a severe acute respiratory syndrome coronavirus 2 (SARS-CoV-2) multi-antigen vaccine co-delivering spike (S), membrane (M), and nucleocapsid (N) antigens elicited significantly more robust humoral and cellular immune responses, as well as superior protective efficacy, compared with the single-antigen vaccines [42]. Chen et al. further demonstrated that multi-antigen combination immunization afforded complete protection against vaccinia virus infection, with significantly higher neutralizing antibody titers than single-antigen immunization, which only conferred partial protection [43]. Our findings in this study are highly consistent with these established conclusions from previous research. Here, the multi-antigen cocktail vaccine composed of VacJ, PtsG, and GAPDH exhibited potent cross-protective activity against two clinically prevalent serotypes of GPS, conferring 80% protection against GPS4 and 100% protection against GPS5 (Figure 2E). Importantly, the immunoprotective performance of the cocktail vaccine was superior to that of any individual antigen component, suggesting the multi-antigen strategy enhanced its cross-protective efficacy.

Furthermore, although the antigen combination VA+PT+PA showed lower protective efficacy than the VA+PT+GA formulation, it still conferred considerable cross-protection against GPS4 and GPS5 challenge (60% and 80%, respectively). This result suggest that GAPDH is more effective than PalA for cross-protection against GPS infection in our model. GAPDH is a core glycolytic enzyme that catalyzes the conversion of glyceraldehyde-3-phosphate to 1, 3 diphosphoglycerate [44]. Beyond its metabolic function, GAPDH also plays a key role in microbial virulence, making it a promising candidate antigen for antibacterial vaccine development [45]. The immunoprotective efficacy of GAPDH has been validated in multiple bacterial pathogens, such as *Listeria monocytogenes*, *Mycobacterium marinum*, *Streptococcus pneumoniae*, and GPS [22, 23, 46, 47]. Notably, recombinant GAPDH has been confirmed to protect against GPS5 infection in both murine and porcine models [23], but its cross-protective efficacy against other prevalent GPS serotypes has not been evaluated prior to our study.

PalA is a peptidoglycan-associated outer membrane lipoprotein with high homology to the P6 protein of *Haemophilus influenzae* [48], a well-characterized protective antigen against *H. influenzae*-induced meningitis [49]. However, previous studies have reported conflicting biological effects of PalA in different bacterial infection models. In an APP vaccine study, PalA exacerbated respiratory symptoms, increased mortality rate, and aggravated lung damage in immunized animals, showing deleterious effects on the protective efficacy of Apx toxin-based subunit vaccines [48]. It has therefore been proposed that PalA is immunogenic but not a protective antigen for APP vaccine development [50]. In contrast, recombinant PalA has been reported to protect against GPS5 infection in a murine model [32]. Indeed, both of the two commercial bivalent subunit vaccines, SS-GPS and PCV2-GPS, utilized PalA as the primary antigen [1]. The drastically opposing protective effect of PalA across different bacterial species warrants further investigation.

VacJ is a conserved outer membrane lipoprotein that has been evaluated as a potential subunit vaccine against GPS infection in both guinea pig and pig models in previous studies [20, 21]. However, these prior studies only focused on the protective effect of VacJ against the single serotype GPS5, and its cross-protective efficacy against other clinically prevalent GPS serotypes has not been assessed to date. In this study, we showed that VacJ immunization not only protected mice from GPS5 infection, consistent with prior work, but also exerted favorable cross-protective efficacy against GPS4 infection in mice. To our knowledge, this is the first report confirming the cross-protective potential of VacJ against GPS4 infection. Furthermore, our study showed that VacJ, as a core component of the multi-antigen cocktail vaccine, synergistically enhanced the broad-spectrum cross-protective efficacy of the formulation when combined with PtsG and GAPDH antigens, further supporting its application value in the development of broadly protective GPS vaccines.

As a type of immune enhancer, adjuvants help reduce the number of immunizations and the amount of antigens, overcome antigen competition in combined vaccines, and importantly, modulate the type of induced immunity [51]. Moreover, many studies have confirmed that the type of adjuvant used in vaccine affect the immune response and protection efficacy of the vaccine against homologous challenge [52–55]. Consequently, we further conducted adjuvant screening following the confirmation of the antigen components for the subunit cocktail vaccine. The results demonstrated that Montanide™ Gel-01 and ISA201 exerted the most robust adjuvant activity in potentiating the immune responses induced by the subunit vaccine (Figure 3A–D). Montanide™ Gel-01 is an aqueous polymeric adjuvant designed for aqueous type vaccines, while Montanide™ ISA201 is a mineral oil based adjuvant that has been developed for the manufacture of water-in-oil-in-water (W/O/W) emulsions [56]. In line with our results, it has been demonstrated that the adjuvant effect of Gel-01 was superior to that of mineral oil and aluminum hydroxide adjuvants for trivalent inactivated GPS serovars 4, 5, and 12 vaccines against Glässer’s disease in terms of antibody titers and protective efficacy [53]. Similar conclusions were also observed for ISA201 adjuvanted foot and mouth disease vaccine [57, 58]. Considering significant difference in body weight changes and bacterial loads in organs of mice after GPS5 challenge, Gel-01 was selected as the optimal adjuvant for the subunit cocktail vaccine in this study.

The magnitude of antigen-specific host immune response after immunization is a core indicator for evaluating the immunogenicity of vaccine candidates. The cocktail vaccine developed in this study induced robust humoral and cellular immune responses in immunized mice (Figure 4). Notably, we observed a significant Th2-skewed immune response elicited by the vaccine. The proportion of splenic CD4⁺ T cells was significantly elevated after immunization compared with CD8⁺ T cells, and the transcriptional upregulation of the Th2 signature cytokine *IL-4* was dramatically stronger than that of the Th1 signature cytokine *IFN-γ* (Figure 4B and G). IL-4 is a key regulator of humoral immunity that drives the proliferation and differentiation of B cells into antibody-secreting plasma cells to produce high levels of antigen-specific IgG, and promotes the differentiation of naïve CD4⁺ T cells into Th2-type effector cells during T cell activation [59]. This Th2-skewed immune response is highly relevant to the protective mechanism against GPS infection. As a typical extracellular bacterial pathogen, the dominant protective immunity against GPS is antibody-mediated humoral immunity. This has been directly confirmed by passive transfer studies, where transfer of anti-GPS immune serum to pigs provided significant protection against Glässer’s disease development [60]. Thus, the Th2-biased response induced by our cocktail vaccine is highly aligned with the core protective mechanism against GPS, as it directly drives the robust antigen-specific antibody production that mediates the observed cross-protective efficacy. These results confirm that the cocktail vaccine preferentially induce a Th2-polarized immune response, which is critical for its protective efficacy against GPS infection.

Notably, no spontaneous death was observed in either the immunized or unimmunized control groups after high virulent GPS5 challenge in piglets. We reasoned that several factors could be attributed to this phenomenon. First of all, we adopted intranasal and intraperitoneal challenge at the same time rather than high-dose intraperitoneal/intravenous challenge only, which causes acute lethal sepsis. Intranasal challenge could mimic the natural respiratory infection route of GPS in commercial pig farms [3], thus we think this model better recapitulates the subacute/chronic Glässer’s disease. Secondly, the piglets were challenged at 10 weeks of age approximately, the peak susceptible age for clinical outbreaks of Glässer’s disease [2, 3]. Compared with 3 to 4-week-old newly weaned piglets, 10-week-old piglets have more complete basal immune function, which may avoid 100% acute lethal outcome and is more consistent with the actual field infection scenario.

In conclusion, a subunit cocktail vaccine was successfully developed against GPS infection in this study. The vaccine showed superior immunogenicity to inactivated vaccine and induced robust humoral and cellular immune responses. Immunization of the vaccine conferred effective cross-protection against GPS4 and GPS5 infections in mice and preliminary protective effects in piglets. These results suggested that the subunit cocktail vaccine established in this study might be a vaccine candidate for preventing GPS infection. Further expanded animal tests and field investigations are necessary to confirm its practical value for the prevention and control of porcine Glässer’s disease.

## Supplementary Information

**Additional file 1. Bacterial strains and plasmids used in this study.**

**Additional file 2. Primers used in this study.**

**Additional file 3. Experimental groups used for the mice experiment in this study.**

**Additional file 4. Gating strategy for the analysis of the proportion of B cells (A) and T cells (B) by flow cytometry at 28 days after immunization.**

**Additional file 5. Prediction of antigenicity of the selected seventeen antigensusing VaxiJen V2.0 server.** The sequences of the antigens PotD (GenBank: ACL31786.1), MnSOD (GenBank: ACL32450.1), GPD (GenBank: ACL32465.1), YgiW (GenBank: ACL33049.1), Omp26 (GenBank: ACL32860.1), QseB (GenBank: ACL33513.1), GAPDH (GenBank: ACL31707.1), TEX (GenBank: ACL32154.1), PalA (GenBank: ACL31779.1), ApbE (GenBank: ACL31801.1), PtsG (GenBank: ACL33546.1), HutZ (GenBank: ACL32674.1), KefA (GenBank: ACL32066.1), VacJ (GenBank: ACL33700.1), ClpP (GenBank: ACL33474.1), CRP (GenBank: ACL33503.1), and QueA (GenBank: ACL32317.1) were retrieved from NCBI database.

**Additional file 6. Antigen cloning and purification.** (A) Cloning of the coding sequences of the selected antigens by PCR. M, marker; 1, *vacj* (687 bp); 2, *ptsg* (366 bp); 3, *pala* (399 bp); 4, *gapdh* (1020 bp); 5, *potd* (981 bp); 6, *mnsod* (639 bp); 7, *ygiw* (705 bp); 8, *gpd* (1503 bp); 9, *abpE* (987 bp); 10, *queA* (1086 bp); 11, *tex* (2300 bp); 12, *hutz* (540 bp); 13, *qseb* (672 bp); 14, *crp* (672 bp); 15, *omp26* (804 bp); 16, *clpp* (582 bp); 17, *hutz* (540 bp); 18, *kefa* (1365 bp). (B) Purification of the selected antigens. M, marker; 1, before IPTG induction; 2, after IPTG induction; 3, supernatant; 4, cell pellets; 5, flow-through; 6, elution by 20 mM imidazole; 7, elution by 50 mM imidazole; 8, elution by 500 mM imidazole.

**Additional file 7. Tertiary structures and molecular docking of VacJ, PtsG, and PalA with TLR2, MHC II, and SLA I.** (A) Predicted tertiary structures of VacJ, PtsG, GAPDH, and PalA by AlphaFold2. (B) Molecular docking of each antigen with immune receptors TLR2, MHC II, and SLA I using the ClusPro 2.0 server. The dashed box denotes the docking region of the complex and the specific amino acid residues involved in docking. (C) The specific docking amino acid residues and the interactions between each amino acid. (D) Interface statistics of the docked complexes.

**Additional file 8. Immune simulation of VacJ, PtsG, GAPDH, and PalA by C-ImmSim server.** (A) The immunoglobulin levels of IgM, IgM+IgG, IgG1, and IgG2; (B) B cell population (active B cell population were in purple); (C) Cytotoxcic T (Tc) cell population (active Tc cell population were in purple); (D) helper T (TH) cell population (active TH cell population were in purple); (E) cytokine levels (IFN-γ and IL-2 levels were presented) in response to vaccination.

**Additional file 9. Identification and reactivity of purified antigens.** (A) Identification of purified recombinant His-tagged antigens VacJ, PalA, GAPDH, and PtsG by western blot using anti-His tag primary antibody. (B) Detection of the antigenicity of the purified antigens with GPS4- and GPS5-positive antiserums. M, marker (Thermo Scientific, 26616). Expected size: VacJ, 43 kDa; GAPDH, 41 kDa; PtsG, 30 kDa; PalA, 35 kDa.

**Additional file 10. Gross necropsy of spleen, lung, and liver of mice with or without immunization.** Spleen, top left; lung, top right; liver, bottom. NC, immunized with PBS plus adjuvant Gel-01, no GPS challenge; PBS+Adjuvant, challenged by GPS4 or GPS5 after immunization with PBS plus adjuvant Gel-01; vaccine, challenged by GPS4 or GPS5 after immunization with the cocktail vaccine; inactivated, challenged by GPS4 or GPS5 after immunization by inactivated vaccine.

**Additional file 11. Changes in body temperature upon immunization with or without the cocktail vaccine.** Body temperatures were recorded every 12 h for a total of 5 days.

**Additional file 12. Clinical symptoms of the piglets after challenge by GPS5.**

**Additional file 13. Gross necropsy observations of the thorax and enterocoelia after GPS5 challenge in piglets.** A–C, control group (PBS+Adjuvant) showing typical characteristics of fibrinous pleuritis, including fibrinous exudation within the thorax, extensive adhesions among the heart, lungs, and thoracic wall (indicated by arrows); D–F, vaccinated group showing minimal adhesions.

**Additional file 14. Clinical, lesion, and bacteriological scores of vaccinated and control piglets challenged by GPS5.**

## Data Availability

All data supporting the findings of this study are available within the paper and its Supplementary Information.

## References

[1] Duan Y, Hao Y, Feng H, Shu J, He Y (2025) Research progress on *Haemophilus parasuis vaccines*. Front Vet Sci 12:1492144 10.3389/fvets.2025.1492144 PMC11851532 40007746

[2] Brockmeier SL, Register KB, Kuehn JS, Nicholson TL, Loving CL, Bayles DO, Shore SM, Phillips GJ (2014) Virulence and draft genome sequence overview of multiple strains of the swine pathogen *Haemophilus parasuis*. PLoS One 9:e103787 10.1371/journal.pone.0103787 PMC4138102 25137096

[3] Aragon V, Segalés J, Tucker AW (2019) Glässer’s disease. Diseases of swine. pp 844–853

[4] Ni HB, Gong QL, Zhao Q, Li XY, Zhang XX (2020) Prevalence of *Haemophilus parasuis* “*Glaesserella parasuis*” in pigs in China: a systematic review and meta-analysis. Prev Vet Med 182:105083 10.1016/j.prevetmed.2020.105083 32652336

[5] Zhang J, Zhao C, Yao M, Qi J, Tan Y, Shi K, Wang J, Zhou S, Li Z (2024) Transcriptome sequencing reveals non-coding RNAs respond to porcine reproductive and respiratory syndrome virus and *Haemophilus parasuis* co-infection in Kele piglets. J Anim Sci Technol 66:663–681 10.5187/jast.2023.e46 PMC11331363 39165737

[6] Gao M, Zuo J, Shen Y, Yuan S, Gao S, Wang Y, Wang Y, Yi L (2023) Modeling co-infection by *Streptococcus suis* and *Haemophilus parasuis* reveals influences on biofilm formation and host response. Animals (Basel) 13:1511 10.3390/ani13091511 PMC10177019 37174548

[7] Sun Q, Yu X, He D, Ku X, Hong B, Zeng W, Zhang H, He Q (2022) Investigation and analysis of etiology associated with porcine respiratory disease complex in China from 2017 to 2021. Front Vet Sci 9:960033 10.3389/fvets.2022.960033 PMC9592729 36304408

[8] Liu H, Xue Q, Zeng Q, Zhao Z (2016) *Haemophilus parasuis* vaccines. Vet Immunol Immunopathol 180:53–58 10.1016/j.vetimm.2016.09.002 27692096

[9] Hendy DA, Haven A, Bachelder EM, Ainslie KM (2023) Preclinical developments in the delivery of protein antigens for vaccination. Expert Opin Drug Deliv 20:367–384 10.1080/17425247.2023.2176844 PMC9992317 36731824

[10] Dai L, Wan J, Zhang R, Xie T, Jia Y, Lu Z, Zhang F, Ke W, Liu F, Lei L (2025) Multi-epitope vaccines Xlc and Ddc against *Glaesserella parasuis* infection in mice. Vet Microbiol 304:110491 10.1016/j.vetmic.2025.110491 40154005

[11] Dai L, Lu Z, Zhang R, Zhang Q, Ming Y, Jia Y, Qi H, He J, Qin X, Ke W, Zhang F, Liu F, Lei L (2026) Evaluation of the cross-immunogenicity and protective efficacy of the multi-epitope antigen Tzi from *Glasserella parasuis* in mouse model. Vaccine 72:128073 10.1016/j.vaccine.2025.128073 41371124

[12] Gong Y, Li N, Li Z, Wang H, Zhang D, Chu H, Lu Z, Jia A, Li F, Lei L (2026) Design of B-cell multi-epitope subunit vaccines against *Glaesserella parasuis* by reverse vaccinology: An *in silico* and *in vivo* study. Transbound Emerg Dis 2026:5696948 10.1155/tbed/5696948 PMC12757848 41488016

[13] Hau SJ, Eberle KC, Nally JE, Nielsen DW, Lippolis JD, Brockmeier SL (2025) Identification of candidate vaccine antigens using 2-D gel electrophoresis and immunoproteomics for cross protection against *Glaesserella parasuis*. Vet Microbiol 307:110594 10.1016/j.vetmic.2025.110594 40516376

[14] An J, Cai J, Zhang B, Li Y (2023) Pili subunit PilA contributes to the cytoadhesion of *Glaesserella parasuis* to host cells and provides immunoprotection. Appl Environ Microbiol 89:e0200222 10.1128/aem.02002-22 PMC10132109 36920203

[15] Huang X, Li Y, Fu Y, Ji Y, Lian K, Zheng H, Wei J, Cai X, Zhu Q (2013) Cross-protective efficacy of recombinant transferrin-binding protein A of *Haemophilus parasuis* in guinea pigs. Clin Vaccine Immunol 20:912–919 10.1128/CVI.00621-12 PMC3675969 23616407

[16] Guizzo JA, Chaudhuri S, Prigol SR, Yu RH, Dazzi CC, Balbinott N, Frandoloso GP, Kreutz LC, Frandoloso R, Schryvers AB (2018) The amino acid selected for generating mutant TbpB antigens defective in binding transferrin can compromise the *in vivo* protective capacity. Sci Rep 8:7372 10.1038/s41598-018-25685-1 PMC5943581 29743502

[17] González-Fernández A, Mencía-Ares O, García-Iglesias MJ, Petrocchi-Rilo M, Miguélez-Pérez R, Perelló-Jiménez A, Herencia-Lagunar E, Acebes-Fernández V, Gutiérrez-Martín CB, Martínez-Martínez S (2024) TbpB-based oral mucosal vaccine provides heterologous protection against Glässer’s disease caused by different serovars of Spanish field isolates of *Glaesserella parasuis*. Porcine Health Manag 10:57 10.1186/s40813-024-00404-7 PMC11606180 39609907

[18] Olvera A, Pina S, Pérez-Simó M, Aragón V, Segalés J, Bensaid A (2011) Immunogenicity and protection against *Haemophilus parasuis* infection after vaccination with recombinant virulence associated trimeric autotransporters (VtaA). Vaccine 29:2797–2802 10.1016/j.vaccine.2011.01.105 21320547

[19] Dai K, Ma X, Yang Z, Chang YF, Cao S, Zhao Q, Huang X, Wu R, Huang Y, Yan Q, Han X, Ma X, Wen X, Wen Y (2019) Polyamine transport protein PotD protects mice against *Haemophilus parasuis* and elevates the secretion of pro-inflammatory cytokines of macrophage via JNK-MAPK and NF-κB signal pathways through TLR4. Vaccines (Basel) 7:216 10.3390/vaccines7040216 PMC6963478 31847381

[20] Li M, Li C, Song S, Kang H, Yang D, Li G (2016) Development and antigenic characterization of three recombinant proteins with potential for Glässer’s disease prevention. Vaccine 34:2251–2258 10.1016/j.vaccine.2016.03.014 26993332

[21] Hau SJ, Luan SL, Loving CL, Nicholson TL, Wang J, Peters SE, Seilly D, Weinert LA, Langford PR, Rycroft AN, Wren BW, Maskell DJ, Tucker AW, Brockmeier SL (2020) Evaluation of the recombinant proteins RlpB and VacJ as a vaccine for protection against *Glaesserella parasuis* in pigs. BMC Vet Res 16:167 10.1186/s12917-020-02377-5 PMC7252510 32460764

[22] Fu S, Zhang M, Ou J, Liu H, Tan C, Liu J, Chen H, Bei W (2012) Construction and immune effect of *Haemophilus parasuis* DNA vaccine encoding glyceraldehyde-3-phosphate dehydrogenase (GAPDH) in mice. Vaccine 30:6839–6844 10.1016/j.vaccine.2012.09.014 23000128

[23] Fu S, Zhang M, Xu J, Ou J, Wang Y, Liu H, Liu J, Chen H, Bei W (2013) Immunogenicity and protective efficacy of recombinant *Haemophilus parasuis* SH0165 putative outer membrane proteins. Vaccine 31:347–353 10.1016/j.vaccine.2012.11.003 23149270

[24] Doytchinova IA, Flower DR (2007) VaxiJen: a server for prediction of protective antigens, tumour antigens and subunit vaccines. BMC Bioinformatics 8:4 10.1186/1471-2105-8-4 PMC1780059 17207271

[25] Krogh A, Larsson B, von Heijne G, Sonnhammer EL (2001) Predicting transmembrane protein topology with a hidden Markov model: application to complete genomes. J Mol Biol 305:567–580 10.1006/jmbi.2000.4315 11152613

[26] Almagro Armenteros JJ, Tsirigos KD, Sønderby CK, Petersen TN, Winther O, Brunak S, von Heijne G, Nielsen H (2019) SignalP 5.0 improves signal peptide predictions using deep neural networks. Nat Biotechnol 37:420–423 10.1038/s41587-019-0036-z 30778233

[27] Kozakov D, Hall DR, Xia B, Porter KA, Padhorny D, Yueh C, Beglov D, Vajda S (2017) The ClusPro web server for protein-protein docking. Nat Protoc 12:255–278 10.1038/nprot.2016.169 PMC5540229 28079879

[28] Laskowski RA, Jabłońska J, Pravda L, Vařeková RS, Thornton JM (2018) PDBsum: structural summaries of PDB entries. Protein Sci 27:129–134 10.1002/pro.3289 PMC5734310 28875543

[29] Rapin N, Lund O, Bernaschi M, Castiglione F (2010) Computational immunology meets bioinformatics: the use of prediction tools for molecular binding in the simulation of the immune system. PLoS One 5:e9862 10.1371/journal.pone.0009862 PMC2855701 20419125

[30] Bak H, Riising HJ (2002) Protection of vaccinated pigs against experimental infections with homologous and heterologous *Haemophilus parasuis*. Vet Rec 151:502–505 10.1136/vr.151.17.502 12430998

[31] Yang Q, Li N, Zheng Y, Tian Y, Liang Q, Zhao M, Chu H, Gong Y, Wu T, Wei S, Wang H, Yan G, Li F, Lei L (2025) Identification and characterization of *ugpE* associated with the full virulence of *Streptococcus suis*. Vet Res 56:82 10.1186/s13567-025-01513-z PMC12001685 40241177

[32] Zhou M, Guo Y, Zhao J, Hu Q, Hu Y, Zhang A, Chen H, Jin M (2009) Identification and characterization of novel immunogenic outer membrane proteins of *Haemophilus parasuis* serovar 5. Vaccine 27:5271–5277 10.1016/j.vaccine.2009.06.051 19576561

[33] Guo L, Xu L, Wu T, Fu S, Qiu Y, Hu CA, Ren X, Liu R, Ye M (2017) Evaluation of recombinant protein superoxide dismutase of *Haemophilus parasuis* strain SH0165 as vaccine candidate in a mouse model. Can J Microbiol 63:312–320 10.1139/cjm-2016-0671 28177794

[34] Yuan F, Fu S, Hu J, Li J, Chang H, Hu L, Chen H, Tian Y, Bei W (2012) Evaluation of recombinant proteins of *Haemophilus parasuis* strain SH0165 as vaccine candidates in a mouse model. Res Vet Sci 93:51–56 10.1016/j.rvsc.2011.04.020 21596404

[35] Mao W, Zhang S, Sun J, Gu J, Xu X, Cai X (2020) Identification of *Haemophilus parasuis* genes uniquely expressed during infection using in vivo-induced antigen technology. Vet Microbiol 243:108650 10.1016/j.vetmic.2020.108650 32273024

[36] Yan X, Gu C, Yu Z, Ding L, He M, Xiao W, Zhao M, Qing Y, He L (2022) Comprehensive analysis of transcriptome and metabolome analysis reveal new targets of *Glaesserella parasuis* glucose-specific enzyme IIBC (PtsG). Microb Pathog 172:105785 10.1016/j.micpath.2022.105785 36150554

[37] Escalante A, Salinas Cervantes A, Gosset G, Bolívar F (2012) Current knowledge of the *Escherichia coli* phosphoenolpyruvate-carbohydrate phosphotransferase system: peculiarities of regulation and impact on growth and product formation. Appl Microbiol Biotechnol 94:1483–1494 10.1007/s00253-012-4101-5 22573269

[38] Vijayakumar V, Vanhove AS, Pickering BS, Liao J, Tierney BT, Asara JM, Bronson R, Watnick PI (2018) Removal of a membrane anchor reveals the opposing regulatory functions of *Vibrio cholerae* glucose-specific enzyme IIA in biofilms and the mammalian intestine. mBio 9:e00858-18 10.1128/mBio.00858-18 PMC6123446 30181246

[39] Khajanchi BK, Odeh E, Gao L, Jacobs MB, Philipp MT, Lin T, Norris SJ (2015) Phosphoenolpyruvate phosphotransferase system components modulate gene transcription and virulence of *Borrelia burgdorferi*. Infect Immun 84:754–764 10.1128/IAI.00917-15 PMC4771366 26712207

[40] Bowden SD, Rowley G, Hinton JC, Thompson A (2009) Glucose and glycolysis are required for the successful infection of macrophages and mice by *Salmonella enterica serovar typhimurium*. Infect Immun 77:3117–3126 10.1128/IAI.00093-09 PMC2708584 19380470

[41] Leekha A, Saeedi A, Sefat K, Kumar M, Martinez-Paniagua M, Damian A, Kulkarni R, Reichel K, Rezvan A, Masoumi S, Liu X, Cooper LJN, Sebastian M, Sands CM, Das VE, Patel NB, Hurst B, Varadarajan N (2024) Multi-antigen intranasal vaccine protects against challenge with sarbecoviruses and prevents transmission in hamsters. Nat Commun 15:6193 10.1038/s41467-024-50133-2 PMC11266618 39043645

[42] Alhashimi M, Sayedahmed EE, Elkashif A, Chothe SK, Wang WC, Murala MST, Gairola V, Wakamatsu N, Gontu A, Ramasamy S, LaBella L, Jakka P, Nair MS, Nissly RH, Kuchipudi SV, Mittal SK (2025) A multi-antigen-based SARS-CoV-2 vaccine provides higher immune responses and protection against SARS-CoV-2 variants. NPJ Vaccines 10:159 10.1038/s41541-025-01198-7 PMC12276352 40683879

[43] Cheng X, Wang Y, Huang B, Bing J, Wang T, Han R, Huo S, Sun S, Zhao L, Shu C, Deng Y, Tan W (2025) Rational mpox vaccine design: immunogenicity and protective effect of individual and multicomponent proteins in mice. Emerg Microbes Infect 14:2482702 10.1080/22221751.2025.2482702 PMC11951338 40105863

[44] Kopeckova M, Pavkova I, Stulik J (2020) Diverse localization and protein binding abilities of glyceraldehyde-3-phosphate dehydrogenase in pathogenic bacteria: the key to its multifunctionality? Front Cell Infect Microbiol 10:89 10.3389/fcimb.2020.00089 PMC7062713 32195198

[45] Perez-Casal J, Potter AA (2016) Glyceradehyde-3-phosphate dehydrogenase as a suitable vaccine candidate for protection against bacterial and parasitic diseases. Vaccine 34:1012–1017 10.1016/j.vaccine.2015.11.072 26686572

[46] Teran-Navarro H, Salcines-Cuevas D, Calderon-Gonzalez R, Tobes R, Calvo-Montes J, Pérez-Del Molino Bernal IC, Yañez-Diaz S, Fresno M, Alvarez-Dominguez C (2021) A comparison between recombinant *Listeria* GAPDH proteins and GAPDH encoding mRNA conjugated to lipids as cross-reactive vaccines for *Listeria*, *Mycobacterium*, and *Streptococcus*. Front Immunol 12:632304 10.3389/fimmu.2021.632304 PMC8092121 33953709

[47] Salcines-Cuevas D, Terán-Navarro H, Calderón-Gonzalez R, Torres-Rodriguez P, Tobes R, Fresno M, Calvo-Montes J, Molino-Bernal I, Yañez-Diaz S, Alvarez-Dominguez C (2021) Glyceraldehyde-3-phosphate dehydrogenase common peptides of *Listeria monocytogenes*, *Mycobacterium marinum* and *Streptococcus pneumoniae* as universal vaccines. Vaccines 9:269 10.3390/vaccines9030269 PMC8002646 33802959

[48] van den Bosch H, Frey J (2003) Interference of outer membrane protein PalA with protective immunity against *Actinobacillus pleuropneumoniae* infections in vaccinated pigs. Vaccine 21:3601–3607 10.1016/s0264-410x(03)00410-9 12922088

[49] Green BA, Metcalf BJ, Quinn-Dey T, Kirkley DH, Quataert SA, Deich RA (1990) A recombinant non-fatty acylated form of the Hi-PAL (P6) protein of *Haemophilus influenzae* elicits biologically active antibody against both nontypeable and type b *H. influenzae*. Infect Immun 58:3272–3278 10.1128/iai.58.10.3272-3278.1990 PMC313649 2401565

[50] Liu C, Cao Y, Yang J, Zhao H, Zahid KR, Zhao J, Qi C, Liu J (2017) Be aware of immunogenic but not protective antigens: the *Actinobacillus pleuropneumoniae* PalA as an example. Protein Pept Lett 24:1059–1065 10.2174/0929866524666170822121558 28828973

[51] Charerntantanakul W (2020) Adjuvants for swine vaccines: mechanisms of actions and adjuvant effects. Vaccine 38:6659–6681 10.1016/j.vaccine.2020.08.054 32888738

[52] Obradovic MR, Corsaut L, Dolbec D, Gottschalk M, Segura M (2021) Experimental evaluation of protection and immunogenicity of *Streptococcus suis* bacterin-based vaccines formulated with different commercial adjuvants in weaned piglets. Vet Res 52:133 10.1186/s13567-021-01004-x PMC8527783 34666827

[53] Xue Q, Zhao Z, Liu H, Chen K, Xue Y, Wang L (2015) First comparison of adjuvant for trivalent inactivated *Haemophilus parasuis* serovars 4, 5 and 12 vaccines against Glässer’s disease. Vet Immunol Immunopathol 168:153–158 10.1016/j.vetimm.2015.11.001 26672914

[54] Oreskovic Z, Nechvatalova K, Krejci J, Kummer V, Faldyna M (2019) Aspects of intradermal immunization with different adjuvants: the role of dendritic cells and Th1/Th2 response. PLoS One 14:e0211896 10.1371/journal.pone.0211896 PMC6370205 30742635

[55] Krejci J, Nechvatalova K, Kudlackova H, Leva L, Bernardy J, Toman M, Faldyna M (2013) Effects of adjuvants on the immune response of pigs after intradermal administration of antigen. Res Vet Sci 94:73–76 10.1016/j.rvsc.2012.07.021 22929313

[56] Petermann J, Bonnefond R, Mermoud I, Rantoen D, Meynard L, Munro C, Lua LHL, Hüe T (2017) Evaluation of three adjuvants with respect to both adverse effects and the efficacy of antibody production to the Bm86 protein. Exp Appl Acarol 72:303–315 10.1007/s10493-017-0156-4 28752480

[57] Dar P, Kalaivanan R, Sied N, Mamo B, Kishore S, Suryanarayana VV, Kondabattula G (2013) Montanide ISA™ 201 adjuvanted FMD vaccine induces improved immune responses and protection in cattle. Vaccine 31:3327–3332 10.1016/j.vaccine.2013.05.078 23735678

[58] Çokçalışkan C, Türkoğlu T, Tuncer-Göktuna P, Sareyyüpoğlu B, Aras-Uzun E, Gündüzalp C, Kürkçü A, Kara O, Karakaya M, Gülyaz V (2021) Evaluation of antibody response of sheep to foot-and-mouth disease vaccine prepared by using different Montanide(TM) oil adjuvants. Trop Biomed 38:154–159 10.47665/tb.38.1.027 33797540

[59] Keegan AD, Leonard WJ, Zhu J (2021) Recent advances in understanding the role of IL-4 signaling. Fac Rev 10:71 10.12703/r/10-71 PMC8442009 34557875

[60] Nedbalcova K, Kucerova Z, Krejci J, Tesarik R, Gopfert E, Kummer V, Leva L, Kudlackova H, Ondriasova R, Faldyna M (2011) Passive immunisation of post-weaned piglets using hyperimmune serum against experimental *Haemophilus parasuis* infection. Res Vet Sci 91:225–229 10.1016/j.rvsc.2010.12.008 21295806

