# Supplementary material for: Development of a cross-protective subunit cocktail vaccine against diverse serotypes of Glaesserella parasuis infection in pigs
Source: Vet Res. 2026 Sep 27;57:172. doi: 10.1186/s13567-026-01846-3 (PMC13617811; doi:10.1186/s13567-026-01846-3)
Supplement: Supplementary file 1 [file 13567_2026_1846_MOESM4_ESM.pdf]

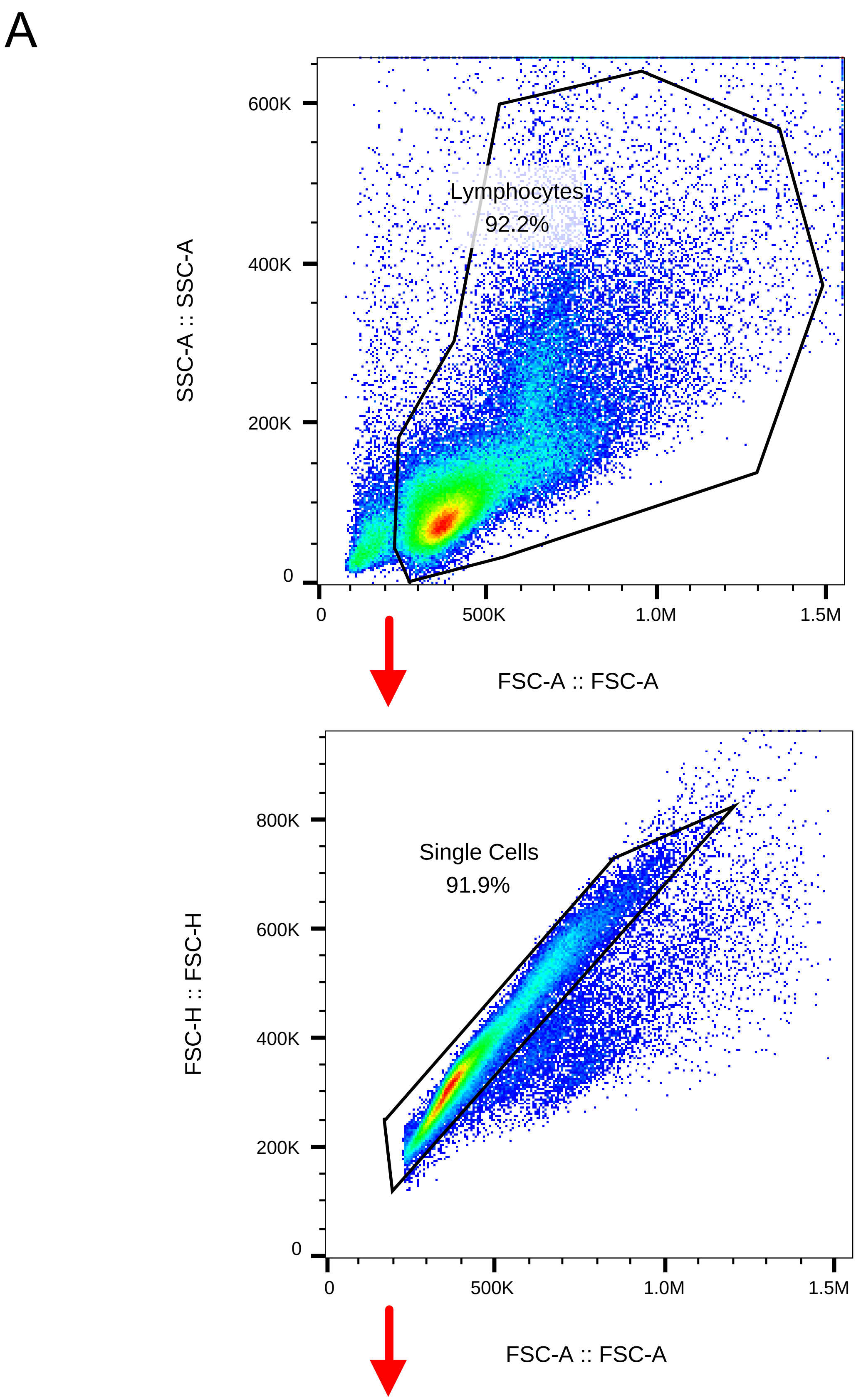
A
Lymphocytes
92.2%
600K
400K
200K
0
SSC-A :: SSC-A
0
500K
1.0M
1.5M
FSC-A :: FSC-A
Single Cells
91.9%
800K
600K
400K
200K
0
FSC-H :: FSC-H
0
500K
1.0M
1.5M
FSC-A :: FSC-A

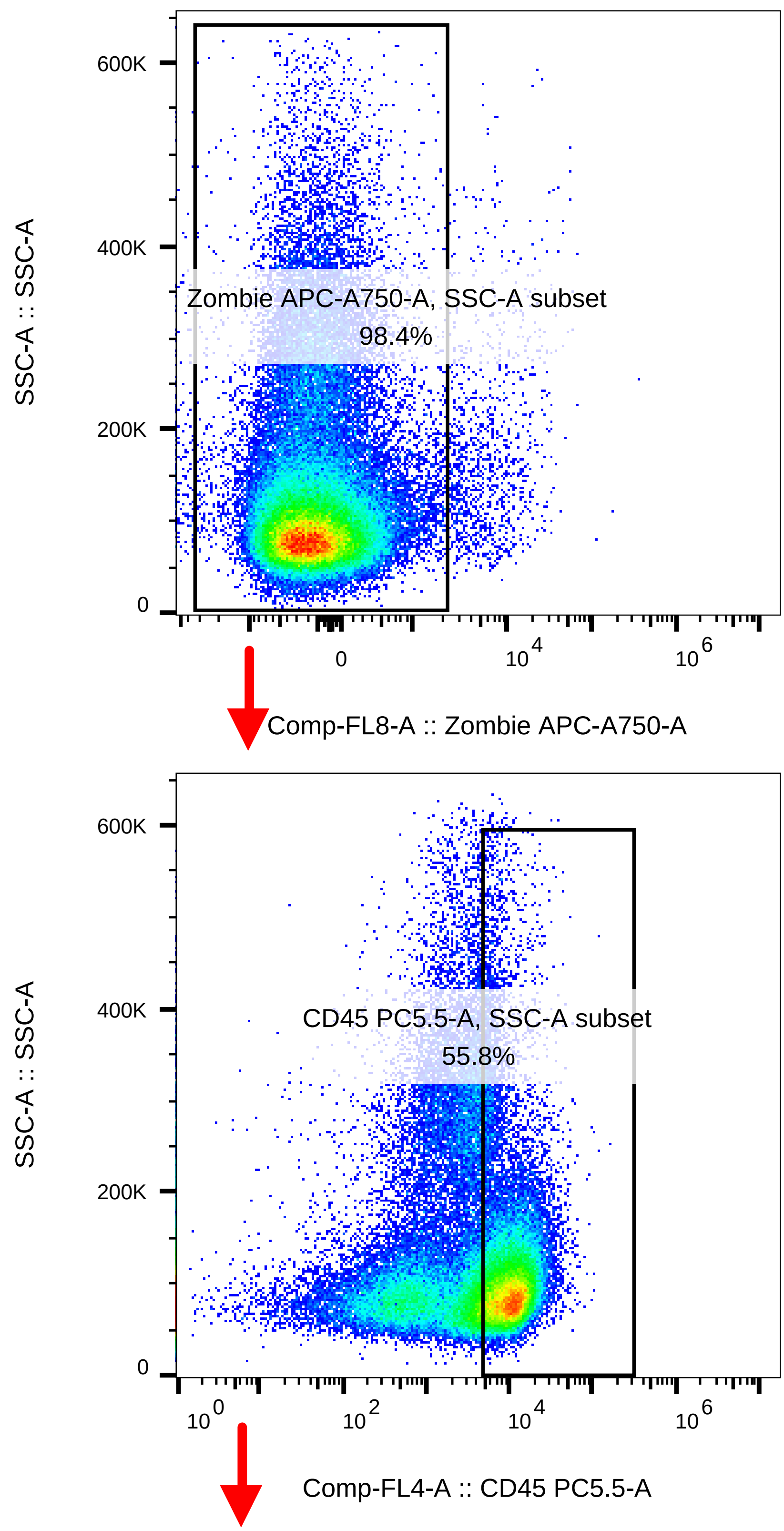

SSC-A :: SSC-A
600K
400K
200K
0
Zombie APC-A750-A, SSC-A subset
98.4%
0
10^4
10^6
Comp-FL8-A :: Zombie APC-A750-A
SSC-A :: SSC-A
600K
400K
200K
0
CD45 PC5.5-A, SSC-A subset
55.8%
10^0
10^2
10^4
10^6
Comp-FL4-A :: CD45 PC5.5-A

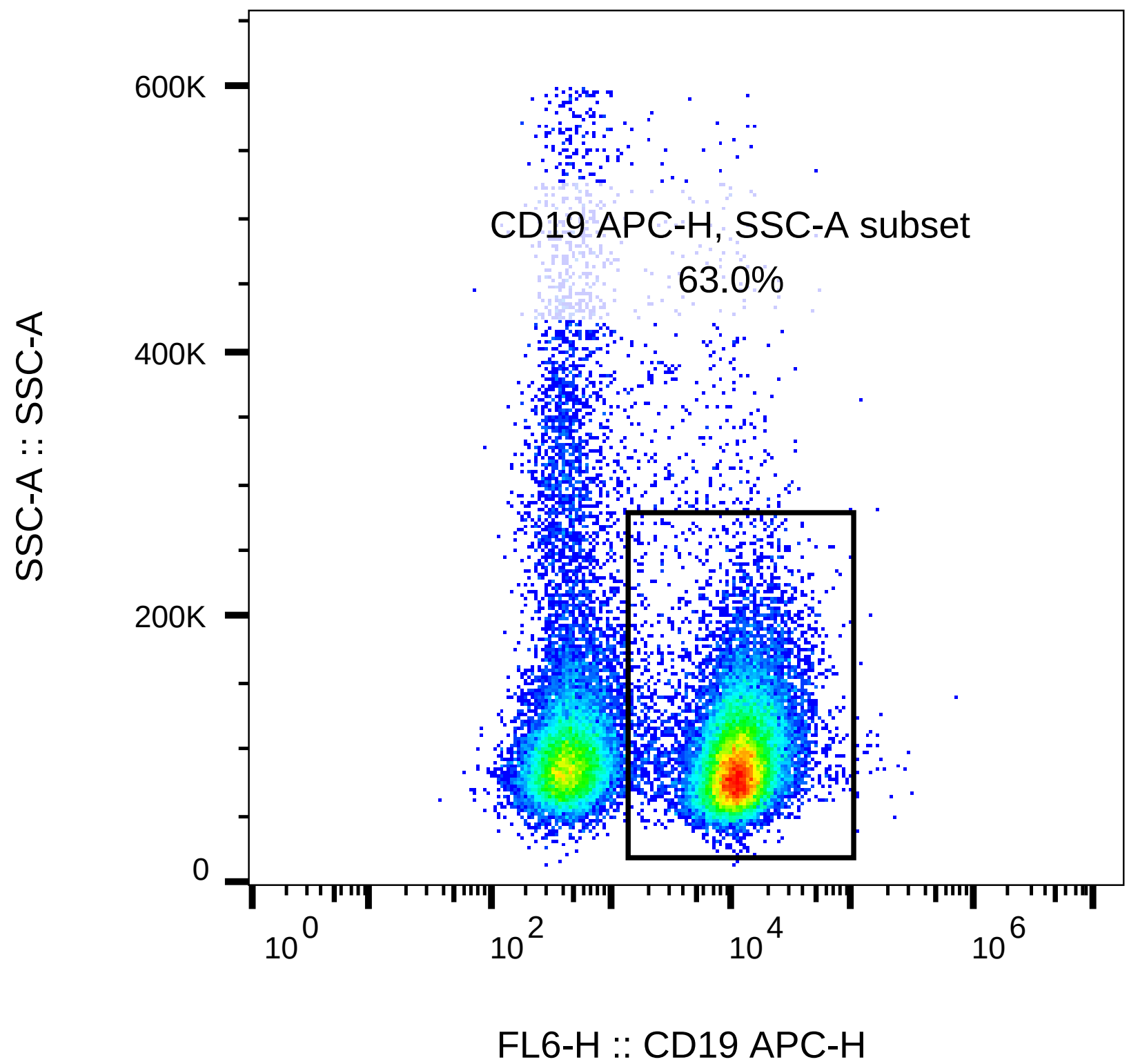

CD19 APC-H, SSC-A subset
63.0%
600K
400K
200K
0
SSC-A :: SSC-A
10^0
10^2
10^4
10^6
FL6-H :: CD19 APC-H

B

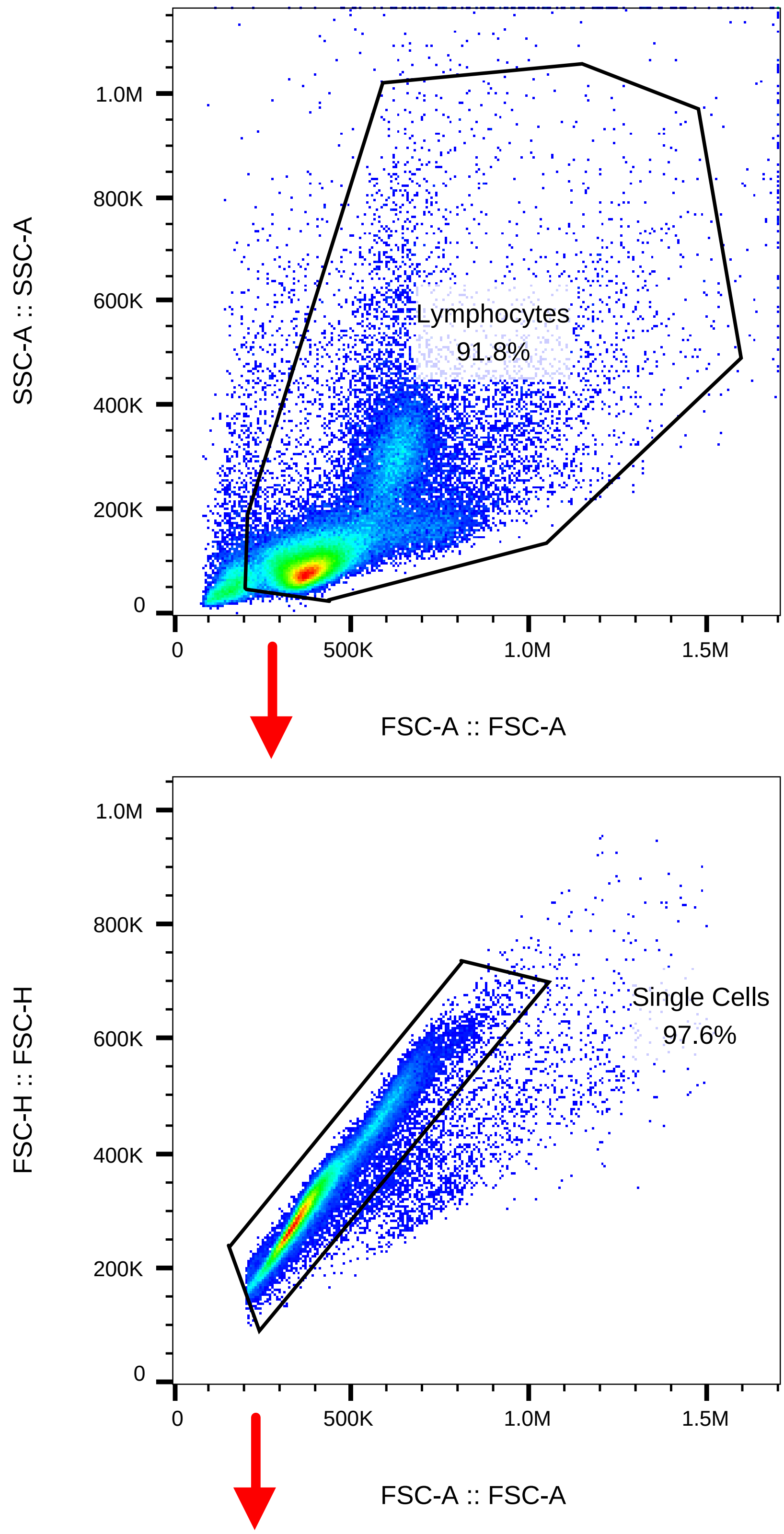

SSC-A :: SSC-A
1.0M
800K
600K
400K
200K
0
Lymphocytes
91.8%
0
500K
1.0M
1.5M
FSC-A :: FSC-A
FSC-H :: FSC-H
1.0M
800K
600K
400K
200K
0
Single Cells
97.6%
0
500K
1.0M
1.5M
FSC-A :: FSC-A

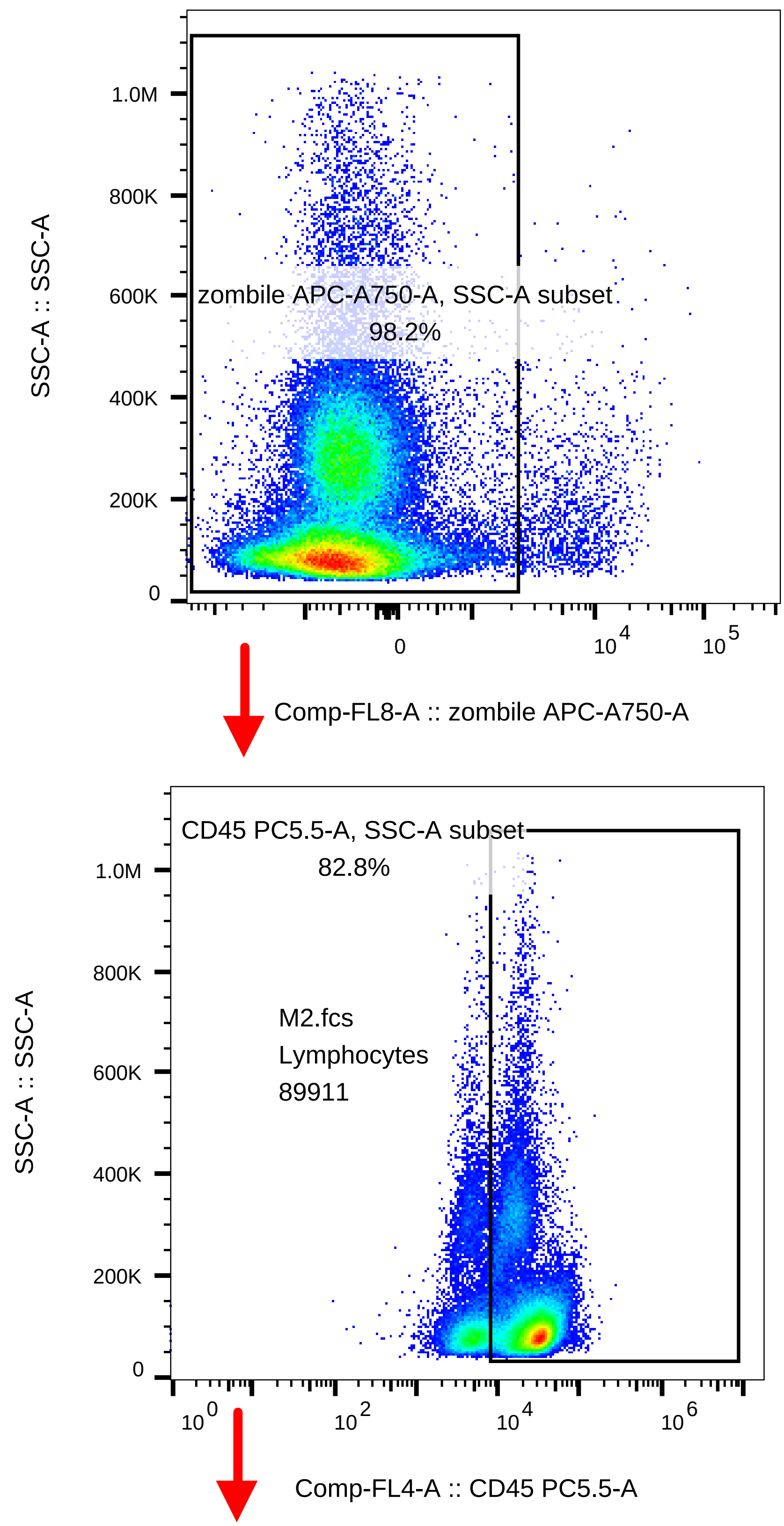

zombile APC-A750-A, SSC-A subset
98.2%
SSC-A :: SSC-A
1.0M
800K
600K
400K
200K
0
0
10^4
10^5
Comp-FL8-A :: zombile APC-A750-A
CD45 PC5.5-A, SSC-A subset
82.8%
M2.fcs
Lymphocytes
89911
SSC-A :: SSC-A
1.0M
800K
600K
400K
200K
0
10^0
10^2
10^4
10^6
Comp-FL4-A :: CD45 PC5.5-A

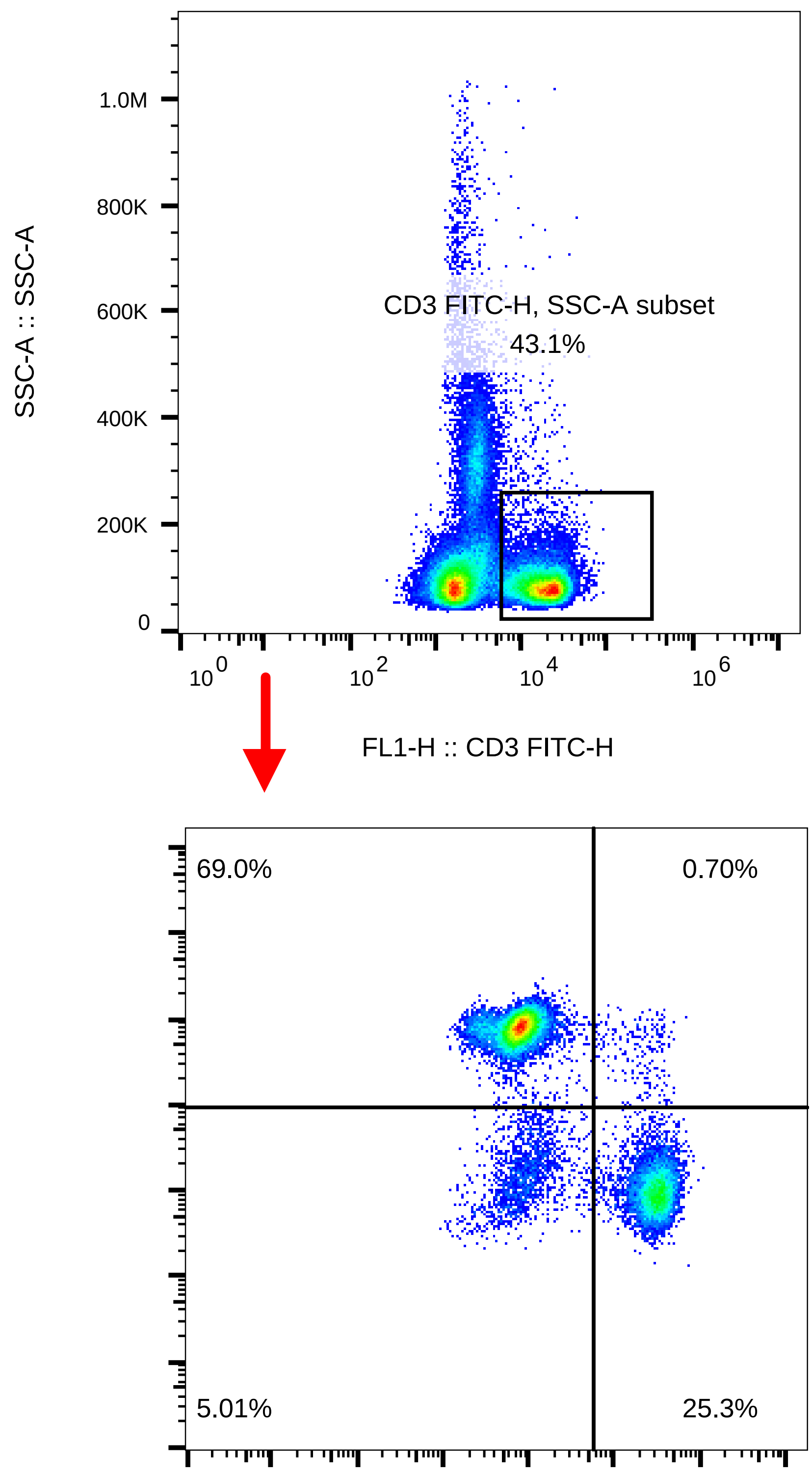

SSC-A :: SSC-A
1.0M
800K
600K
400K
200K
0
CD3 FITC-H, SSC-A subset
43.1%
10^0
10^2
10^4
10^6
FL1-H :: CD3 FITC-H
69.0%
0.70%
5.01%
25.3%
