# Supplementary material for: Development of a cross-protective subunit cocktail vaccine against diverse serotypes of Glaesserella parasuis infection in pigs
Source: Vet Res. 2026 Sep 27;57:172. doi: 10.1186/s13567-026-01846-3 (PMC13617811; doi:10.1186/s13567-026-01846-3)
Supplement: Supplementary file 2 [file 13567_2026_1846_MOESM12_ESM.docx]

**Additional file 12.** Clinical symptoms of the piglets after challenge by GPS5.

| Groups | DPI^1^ | ABT^2^ | MS^3^ |  | Breath | Posture | Food intake |
| --- | --- | --- | --- | --- | --- | --- | --- |
| PBS + Adjuvant | 1 | 39.4 | depressed |  | normal | normal | normal |
|  | 2 | 40.1 | depressed |  | normal | lie down | normal |
|  | 3 | 40.2 | depressed |  | ND^4^ | lie down | decreased |
|  | 4 | 40.2 | depressed |  | ND^4^ | lie down | decreased |
|  | 5 | 39.5 | depressed |  | ND^4^ | lie down | decreased |
|  | 6 | 39.6 | normal |  | ND^4^ | normal | normal |
| Vaccine | 1 | 39.1 | normal |  | normal | lie down | normal |
|  | 2 | 40.0 | depressed |  | normal | lie down | decreased |
|  | 3 | 39.8 | depressed |  | ND^4^ | lie down | decreased |
|  | 4 | 39.0 | normal |  | ND^4^ | normal | increased |
|  | 5 | 39.1 | normal |  | normal | normal | increased |
|  | 6 | 39.3 | normal |  | normal | normal | normal |

^1^DPI, days post infection. ^2^ABT, average body temperature (℃). ^3^MS, mental status. ^4^ND, nasal discharge.
