# Supplementary material for: Development of a cross-protective subunit cocktail vaccine against diverse serotypes of Glaesserella parasuis infection in pigs
Source: Vet Res. 2026 Sep 27;57:172. doi: 10.1186/s13567-026-01846-3 (PMC13617811; doi:10.1186/s13567-026-01846-3)
Supplement: Supplementary file 3 [file 13567_2026_1846_MOESM14_ESM.docx]

**Additional file 14.** Clinical, lesion and bacteriological scores of vaccinated and control piglets challenged by GPS5.

| Groups | Survival | Clinical score^1^ | Lesion score^2^ |  | Bacteriological score^3^ | Total score |
| --- | --- | --- | --- | --- | --- | --- |
| PBS + Adjuvant | Yes | 6 | 3 |  | 2 | 11 |
|  | Yes | 6 | 3 |  | 2 | 11 |
|  | Yes | 5 | 3 |  | 2 | 10 |
| Vaccine | Yes | 5 | 0 |  | 1 | 6 |
|  | Yes | 3 | 0 |  | 1 | 4 |
|  | Yes | 0 | 0 |  | 1 | 1 |
|  |  |  |  |  |  |  |

^1^Clinical score is the sum of the other scores for temperature, general condition, limping and other abnormalities. Temperature: 0, <40.0; 1, 40.0 to 40.4; 2, 40.5 to 40.9; 3, 41.0 to 41.4; 4, 41.5 to 41.9; 5, 42.0 to 42.4; 6, > 42.4. General condition: 0, normal behavior; 1, does not eat; 2, does not eat and depressed; 3, depressed and lies down; 4, moribund. Limping: 0, no limping; 1, abnormal gait on one leg; 2, limping on one leg; 3, limping on more than one leg; 4, springhalt; 6, unable to walk. Other abnormalities: 0, no abnormalities; 1, minor abnormalities; 2, severe abnormalities.

^2^0, no macroscopic lesions; 1, status after fibrinous/fibrous peritonitis/pleuritis/pericarditis; 2, mild chronic fibrinous/fibrous peritonitis/pleuritis/pericarditis or increased amount of synovia; 3, severe fibrinous or mild fibrinopurulent serositis in at least one of the following cavities: abdomen, pleura, pericardium or joint; 4, severe fibrinous or mild fibrinopurulent serositis in more than one of the aforementioned cavities; 5, moderate to severe fibrinopurulent serositis in at least one of the cavities; 6, moderate to severe fibrinopurulent serositis in more than one of the cavities.

^3^0, no isolations of GPS5; 1, few colonies of GPS5; 2, abundant growth of GPS5.
