# Supplementary material for: Development of a cross-protective subunit cocktail vaccine against diverse serotypes of Glaesserella parasuis infection in pigs
Source: Vet Res. 2026 Sep 27;57:172. doi: 10.1186/s13567-026-01846-3 (PMC13617811; doi:10.1186/s13567-026-01846-3)
Supplement: Supplementary file 4 [file 13567_2026_1846_MOESM1_ESM.docx]

**Additional file 1.** Bacterial strains and plasmids used in this study. Amp^r^, ampicillin resistance. Kana^r^, kanamycin resistance.

| Bacterial strains | Description^a^ | Source |
| --- | --- | --- |
| GPS4 | *G. parasuis* serotype 4 | This study |
| GPS5 | *G. parasuis* serotype 5 strain Nagasaki | This study |
| *E. coli* DH5α | λφ80d*lacZ* ΔM15 Δ(*lacZYA*-argF)U169  *recA1* *endA1 hsdR17*(rK- mK- ) *supE44*  *thi-1 gyrA relA1* | New England Biolabs |
| *E. coli* BL21 (DE3) | F^-^ *ompT hsdSB*(r^-^_B_m^-^_B_) *gal dcm* (DE3) | Novagen |
| Plasmids | |  |
| pET32a | Expression vector with T7 promoter, ^a^Amp^r^ | Novagen |
| pET28a | Expression vector with T7 promoter, ^b^Kana^r^ | Novagen |
| pET32a::potd | pET32a inserted with synthesized *potd*, Amp^r^ | This study |
| pET32a::mnsod | pET32a inserted with synthesized *mnsod*, Amp^r^ | This study |
| pET32a::gpd | pET32a inserted with synthesized gpd, Amp^r^ | This study |
| pET32a::ygiw | pET32a inserted with *ygiw*, Amp^r^ | This study |
| pET32a::omp26 | pET32a inserted with *omp26*, Amp^r^ | This study |
| pET32a::crp | pET32a inserted with *crp*, Amp^r^ | This study |
| pET32a::vacj | pET32a inserted with *vacj*, Amp^r^ | This study |
| pET32a::ptsg | pET32a inserted with *ptsg*, Amp^r^ | This study |
| pET32a::qseb | pET32a inserted with *qseb*, Amp^r^ | This study |
| pET32a::kefa | pET32a inserted with *kefa*, Amp^r^ | This study |
| pET32a::hutz | pET32a inserted with *hutz*, Amp^r^ | This study |
| pET28a::pala | pET28a inserted with *pala*, Kana^r^ | This study |
| pET28a::tex | pET28a inserted with *tex*, Kana^r^ | This study |
| pET28a::apbe | pET28a inserted with *apbe*, Kana^r^ | This study |
| pET28a::clpp | pET28a inserted with *clpp*, Kana^r^ | This study |
| pET32a::quea | pET32a inserted with *quea*, Amp^r^ | This study |
| pET28a::gapdh | pET28a inserted with *gapdh*, Kana^r^ | This study |

^a^Amp^r^, ampicillin resistance; ^b^Kana^r^, kanamycin resistance
