# Supplementary material for: Development of a cross-protective subunit cocktail vaccine against diverse serotypes of Glaesserella parasuis infection in pigs
Source: Vet Res. 2026 Sep 27;57:172. doi: 10.1186/s13567-026-01846-3 (PMC13617811; doi:10.1186/s13567-026-01846-3)
Supplement: Supplementary file 5 [file 13567_2026_1846_MOESM2_ESM.docx]

**Additional file 2**. Primers used in this study.

| Primers | Sequence (5’-3’) |
| --- | --- |
| Gene cloning | |
| MnSOD-F | GCGGGATCCATGGCATACACATTACC |
| MnSOD-R | GCGCTCGAGTTATGCTTGGGATTCAAAA |
| PotD-F | CGCGGATCCCATCTTTATACTTGGACAGA |
| PotD-R | CCGCTCGAGTTACTTCGCAGCTTTTAACT |
| GPD-F | GCGGGATCCATGAAAGCAGACAATAATTG |
| GPD-R | GCGCTCGAGTTACACTTTTTTCTTCATCAA |
| PalA-F | GCGGGATCCATGAAAAAACTAGCTAAAG |
| PalA-R | GCGCTCGAGTTAGTACTCTAACACTGCA |
| YgiW-F | GCGGGATCCATGACATTGAAAAAATATT |
| YgiW-R | GCGCTCGAGTTATCTTTCAAATTTCACC |
| QseB-F | GCGGATCCATGCGTATTTTATTAATTGAAGAC |
| QseB-R | CGCCTCGAGTTAAGCAACTTCATCGTTTTT |
| GAPDH-F | GCGGATCCATGGCAATTAAAATTG |
| GAPDH-R | GCCTCGAGTTAGCCTTTGTAGTTG |
| Omp26-F | CCGGATCCAAAAATTTATTTAAACTTGC |
| Omp26-R | GGCTCGAGTTTTTCACTTCTTCTGG |
| PtsG-F | GCGGATCCATGTTCAAATTTATTAAAAACCTG |
| PtsG-R | CGCCTCGAGTTTTTTTAAAATAGCATTAATCGC |
| VacJ-F | GCGGATCCACCTCTTCTATTGATCCTGAAACTG |
| VacJ-R | CGCCTCGAGATCAATATTTTTTAGCTGTTCTTC |
| TEX-F | GCGGATCCATGACAGAACTCAACCACCAA |
| TEX-R | CGCCTCGAGCTTCTTCCACCCCTTCAAC |
| HutZ-F | GCGGATCCATGTCAAATCGTCAAGAAGTATTAC |
| HutZ-R | CGCCTCGAGTGCCTCTTTCTCGTGCGGAC |
| ApbE-F | GCGGATCCAATAAAGAACCCCAACAAATTGC |
| ApbE-R | CGCCTCGAGTTTTTGATTAATTAATTTTTCAAAGGC |
| KefA-F | GCGGATCCGTGGAAGATTTTGTGTCTGCG |
| KefA-R | CGCCTCGAGGTTCTGCCCCAAATTTTCAAAG |
| CRP-F | GCGGATCCATGCAAGATGTTTCAATCTCAACC |
| CRP-R | CGCCTCGAGTCTTGTCCCATACACAACGAT |
| ClpP-F | GCGGATCCATGGCTGTAATCCCTATGGTC |
| ClpP-R | CGCCTCGAGTCTCTTTGTTACAACGTCATCAATTAA |
| QueA-F | GCGGATCCGTGCGTGTAACCGATTTCC |
| QueA-R | CGCCTCGAGGGGAACATCCCCCAACGC |
| qRT-PCR primers | |
| IL-6-F | CTTCTTGGGACTGATGCTGGTGAC |
| IL-6-R | TCTGTTGGGAGTGGTATCCTCTGTG |
| TNF-α-F | CCCTCACACTCACAAACCAC |
| TNF-α-R | ACAAGGTACAACCCATCGGC |
| IL-1β-F | TGGACCTTCCAGGATGAGGACA |
| IL-1β-R | GTTCATCTCGGAGCCTGTAGTG |
| GAPDH-F | TGAGCCTCCTCCAATTCAACC |
| GAPDH-R | AAATCCGTTCACACCGACCT |
| IFN-γ-F | CTGGAGGAACTGGCAAAAGGATGG |
| IFN-γ-R | GACGCTTATGTTGTTGCTGATGGC |
| IL-2 F | TCCTCTCCAGGGAGCTAACA |
| IL-2 R | ATTCCAAAACCAGCAACCCATC |
| IL-4 F | TCTTGATAAAACTTAATTGTCTCTCGTCAC |
| IL-4 R | GCAGGATGACAAACTAGCTGGG |
