# Supplementary material for: Development of a cross-protective subunit cocktail vaccine against diverse serotypes of Glaesserella parasuis infection in pigs
Source: Vet Res. 2026 Sep 27;57:172. doi: 10.1186/s13567-026-01846-3 (PMC13617811; doi:10.1186/s13567-026-01846-3)
Supplement: Supplementary file 6 [file 13567_2026_1846_MOESM3_ESM.docx]

**Additional file 3**. Experimental groups used for the mice experiment in this study.

|  | Experimental groups | Ingredients (200 μL) | Number of mice | Challenging strain |
| --- | --- | --- | --- | --- |
| Selection of protective  single antigens | QseB | 100 μg + Gel-01 (10%) | 10/group | *Glaesserella parasuis* serotype 4 /serotype5  (GPS4/GPS5) |
|  | Omp26 |  |  |  |
|  | GAPDH |  |  |  |
|  | TEX |  |  |  |
|  | PalA |  |  |  |
|  | ApbE |  |  |  |
|  | YgiW |  |  |  |
|  | KefA |  |  |  |
|  | HutZ |  |  |  |
|  | PtsG |  |  |  |
|  | MnSOD |  |  |  |
|  | VacJ |  |  |  |
|  | ClpP |  |  |  |
|  | QueA |  |  |  |
|  | PBS + Adjuvant | 200 µL PBS with Gel-01 (10%) |  |  |
| Selection of protective  antigen combinations | VA + PT | 50 μg + 50 μg + Gel-01 (10%) | 10/group |  |
|  | VA + PT + PA | 33 μg + 33 μg + 33 μg Gel-01 (10%) |  |  |
|  | VA + PT + GA |  |  |  |
|  | VA + PA + GA |  |  |  |
|  | PT + GA + PA |  |  |  |
|  | Inactivated vaccine | 200 µL inactivated vaccine (Kefuning, China) |  |  |
|  | PBS + Adjuvant | 200 µL PBS with Gel-01 (10%) |  |  |
| Selection of optimal adjuvants | Gel-01 | PBS with 10% Gel-01 | 10/group |  |
|  | ISA201 | PBS with 10% ISA201 |  |  |
|  | ISA206 | PBS with 10% ISA206 |  |  |
|  | ISA563 | PBS with 10% ISA563 |  |  |
|  | OIW | PBS with OIW |  |  |
|  | Aluminum | PBS with aluminum |  |  |
|  | PBS | 200 µL PBS |  |  |
