# Supplementary material for: Development of a cross-protective subunit cocktail vaccine against diverse serotypes of Glaesserella parasuis infection in pigs
Source: Vet Res. 2026 Sep 27;57:172. doi: 10.1186/s13567-026-01846-3 (PMC13617811; doi:10.1186/s13567-026-01846-3)
Supplement: Supplementary file 7 [file 13567_2026_1846_MOESM5_ESM.docx]

**Additional file 5.** Prediction of antigenicity of the selected seventeen antigens using VaxiJen V2.0 server. The sequences of the antigens PotD (GenBank: ACL31786.1), MnSOD (GenBank: ACL32450.1), GPD (GenBank: ACL32465.1), YgiW (GenBank: ACL33049.1), Omp26 (GenBank: ACL32860.1), QseB (GenBank: ACL33513.1), GAPDH (GenBank: ACL31707.1), TEX (GenBank: ACL32154.1), PalA (GenBank: ACL31779.1), ApbE (GenBank: ACL31801.1), PtsG (GenBank: ACL33546.1), HutZ (GenBank: ACL32674.1), KefA (GenBank: ACL32066.1), VacJ (GenBank: ACL33700.1), ClpP (GenBank: ACL33474.1), CRP (GenBank: ACL33503.1), and QueA (GenBank: ACL32317.1) were retrieved from NCBI database.

| **Antigens** | **PotD** | **MnSOD** | | **GPD** | **YgiW** | **Omp26** | **QseB** | **GAPDH** |
| --- | --- | --- | --- | --- | --- | --- | --- | --- |
| **VaxiJen score** | 0.3103 | 0.4995 | 0.3270 | | 0.4885 | 0.5328 | 0.4273 | 0.6018 |
| **Antigens** | **TEX** | **PalA** | **ApbE** | | **PtsG** | **HutZ** | **KefA** | **VacJ** |
| **VaxiJen score** | 0.4574 | 0.7039 | 0.5503 | | 0.4529 | 0.4084 | 0.4371 | 0.4913 |
| **Antigens** | **ClpP** | **CRP** | **QueA** | |  |  |  |  |
| **VaxiJen score** | 0.4254 | 0.3163 | 0.4707 | |  |  |  |  |
